# Crosstalk between cancer cells and tumor associated macrophages is required for mesenchymal circulating tumor cell-mediated colorectal cancer metastasis

**DOI:** 10.1186/s12943-019-0976-4

**Published:** 2019-03-30

**Authors:** Chen Wei, Chaogang Yang, Shuyi Wang, Dongdong Shi, Chunxiao Zhang, Xiaobin Lin, Qing Liu, Rongzhang Dou, Bin Xiong

**Affiliations:** 1 Department of Gastrointestinal Surgery & Department of Gastric and Colorectal Surgical Oncology, Zhongnan Hospital of Wuhan University, Wuhan, China; 2 Hubei Key Laboratory of Tumor Biological Behaviors, Wuhan, China; 3 Hubei Cancer Clinical Study Center, Wuhan, China

**Keywords:** Tumor-associated macrophages, Colorectal cancer, EMT, Circulating tumor cell, Metastasis, Prognosis

## Abstract

**Background:**

Tumor-associated macrophages (TAMs) are major components of tumor microenvironment that frequently associated with tumor metastasis in human cancers. Circulating tumor cell (CTC), originating from primary tumor sites, is considered to be the precursors of tumor metastasis. However, the regulatory mechanism of TAMs in CTC-mediated tumor metastasis still remains unclear.

**Methods:**

Immunohistochemical staining was used to detect the macrophages infiltration (CD68 and CD163), epithelial–mesenchymal transition (EMT) markers (E-cadherin and Vimentin) expression in serial sections of human colorectal cancer (CRC) specimens. Then, the correlations between macrophages infiltration and clinicopathologic features, mesenchymal CTC ratio, and patients’ prognosis were analyzed. A co-culture assay in vitro was used to evaluate the role of TAMs on CRC EMT, migration and invasion, and ELISA, luciferase reporter assay and CHIP were performed to uncover the underlying mechanism. Furthermore, an in vivo model was carried out to confirm the effect of TAMs on mesenchymal CTC-mediated metastasis.

**Results:**

Clinically, CD163^+^ TAMs infiltrated in invasive front was associated with EMT, mesenchymal CTC ratio, and poor prognosis in patients with CRC. CRC–conditioned macrophages regulated EMT program to enhance CRC cells migration and invasion by secreting IL6. TAMs-derived IL6 activated the JAK2/STAT3 pathway, and activated STAT3 transcriptionally inhibited the tumor suppressor miR-506-3p in CRC cells. miR-506-3p, a key miRNA regulating FoxQ1, was downregulated in CRC cells, resulting in increased FoxQ1 expression, which in turn led to the production of CCL2 that promoted macrophage recruitment. Inhibition of CCL2 or IL6 broke this loop and reduced macrophage migration and mesenchymal CTC-mediated metastasis, respectively.

**Conclusions:**

Our data indicates that TAMs induce EMT program to enhance CRC migration, invasion, and CTC-mediated metastasis by regulating the JAK2/STAT3/miR-506-3p/FoxQ1 axis, which in turn leads to the production of CCL2 that promote macrophage recruitment, revealing a new cross-talk between immune cells and tumor cells in CRC microenvironment.

**Electronic supplementary material:**

The online version of this article (10.1186/s12943-019-0976-4) contains supplementary material, which is available to authorized users.

## Background

Colorectal cancer (CRC) is the third most common malignancy and the second leading cause of cancer-related mortality worldwide [1]. Metastasis, a multi-step complex process involving multiple factors, is still the main cause for CRC-related deaths [2]. Circulating tumor cell (CTC), originating from primary tumor or metastatic sites, is considered to be the precursors of metastases [3]. Previously, our group reported several methods for CTC capture and identification, and demonstrated CTCs detection was closely associated with multiple clinicopathological factors that predicted high metastatic risk in different solid cancers, including gastric, colorectal and hepatocellular cancer [4–7]. Subsequently, we found that only quantifying the CTC count is not sufficient to explain the important role of CTC in the metastasis process, nor can it understand the mechanisms of CTC-mediated metastasis. Meanwhile, we also found that CTC could undergo epithelial-mesenchymal transition (EMT). Moreover, numerous studies demonstrated mesenchymal CTC (^M^CTC) had more prognostic value than total CTC, which was positively associated with tumor progression and poor patient’s survival in CRC, and knowing about the phenotype traits of CTC could give more information about CRC development [8, 9]. Currently, EMT in cancer, as known to increase cell motility and invasive potential, has been proposed to play the critical role in CTC generation [10]. CTC, which gains more mesenchymal traits by EMT, is easy to survive and metastasize [11, 12]. Therefore, exploring the underlying mechanisms of CTC EMT have great significance for further understanding the metastatic process in CRC.

Tumor microenvironment (TME) represents the necessary prerequisite for cancer progression and metastasis [13]. Macrophages in the TME, referred to as tumor-associated macrophages (TAMs), are one of the most abundant types of cells, and exhibit different phenotypes and functions in response to various microenvironmental signals generated from tumor and stromal cells [14]. At present, numerous studies have showed that the localization and density of TAMs are associated with poor clinical outcome in several kinds of solid cancers, including bladder, breast, renal, prostate and gastric cancer [15–19]. In terms of CRC, the exact roles of TAMs are seem to be somewhat contradictory [20, 21]. Noteworthy, emerging studies have suggested that TAMs play important roles in tumor metastases by regulating EMT of cancer cells. In hepatocellular carcinoma (HCC), HCC-derived IL-8 stimulated the M2 polarization of TAMs, which promoted the EMT and invasive potential of HCC cells [22]. Additionally, Wang and colleagues revealed that pancreatic cancer (PC) cells activated macrophages to the M2 phenotype, which then promoted EMT progress to increase migration and invasion of PC cells [23]. Reciprocally, Su et al. showed that cancer cells that have undergone EMT secreted GM-CSF to promote macrophages recruitment, thereby mediating breast cancer metastasis [24]. However, the roles and mechanisms of the crosstalk between TAMs and cancer cells in EMT of CRC are still unclear.

Given the crucial roles of TAMs, EMT and CTC in dictating CRC metastasis, we speculated that the crosstalk between TAMs and tumor cells could promote ^M^CTC-mediated tumor metastasis by regulating the EMT program. In the present study, our results showed that CD163^+^ TAMs at invasive front were significantly correlated with EMT status, ^M^CTC ratio, and patients’ prognosis in CRC. In vitro and in vivo experimental evidences also showed a significant increase in tumor EMT to enhance migration, invasion and metastasis in the presence of TAMs, confirming their pro-tumor functions in CRC. Further mechanistic studies revealed that TAMs induce EMT in CRC cells by regulating the STAT3/miR-506-3p/FoxQ1 axis, ﻿which in turn lead to the production of CCL2 to favor macrophages recruitment. These findings demonstrate a positive feedback loop between cancer cells and TAMs promotes CRC metastasis by regulating the EMT program of CTC, contributing to new insight concerning TME and CRC progression.

## Methods

### Patients and tissue samples

Primary CRC tissue samples were obtained from 81 patients who underwent curative resection at Zhongnan Hospital of Wuhan University (Wuhan, China). All included patients were identified as adenocarcinoma of colorectal by histopathology and had available preoperative CTC and survival data. Moreover, all patients were devoid of neoadjuvant chemotherapy or radiotherapy before surgical resection and did not be diagnosed with autoimmune diseases. Peripheral blood (PB) samples with a volume of 2.5 ml from all patients were collected in EDTA-containing tubes (BD, USA) at the time of one day before surgery. Formalin-fixed, paraffin-embedded (FFPE) cancer tissue specimens were obtained from these patients after surgery. All samples were collected with informed consent from patients, and all related procedures were performed with the approval of the internal review and ethics boards of Zhongnan Hospital of Wuhan University.

### Immunohistochemistry

Paraffin-embedded samples were serially sectioned at 4 μm thickness. Antigen retrieval was performed by a pressure cooker for 30 min in 0.01 M citrate buffer (pH 6.0), followed by treatment with 3% hydrogen peroxide for 5 min. Specimens were incubated with monoclonal antibodies against human CD68 (1:500; Abcam, USA), CD163 (1:50; Abcam, USA), E-cadherin (1:200; CST, USA), Vimentin (1:200; CST, USA), IL6 (1:100; CST, USA) and FoxQ1 (1:100; Sigma-Aldrich, USA) overnight at 4 degree. Immunostaining was performed using DAB or Permanent Red (Dako) according to the manufacturer’s instructions. For negative control, isotype-matched antibodies were applied. Cells stained with indicated antibody were calculated calculated per field of view, with at least 10 view-fields per section were evaluated at 400× magnification. The expression levels of CD68, CD163, E-cadherin, Vimentin, IL6 and FoxQ1 were scored semiquantitatively based on staining intensity and distribution using the immunoreactive score (IRS) as described elsewhere [25, 26]. Briefly, Immunoreactive score (IRS) = SI (staining intensity) × PP (percentage of positive cells). SI was assigned as: 0 = negative; 1 = weak; 2 = moderate; 3 = strong. PP is defined as 0 = 0%; 1 = 0–25%; 2 = 25–50%; 3 = 50–75%; 4 = 75–100%. All of the included patients were dichotomized into two groups (high expression group: >median score; low expression group: ≤median score) based on the median score of CD68 and CD163 expression.

### CTC isolation and identification

CTC was enriched using the CTCBIOPSY^®^ device (Wuhan YZY Medical Science and Technology Co., Ltd., Wuhan, China) as described in our previous study [7]. The samples were processed according to the manufacturer’s instructions. In brief, 2.5 ml blood sample of included patient was diluted up to 8 ml with 0.9% physiological saline containing 0.2% paraformaldehyde and left for 10 min at room temperature, then transferred to ISET tubes with an 8 μm diameter aperture membrane. After filtered by positive pressure from 12 mmHg to 20 mmHg, candidate CTC was adhered to the membrane and were identified by three-color immunofluorescence staining. Immunofluorescence staining was performed as described in our previous study [7]. In brief, membranes with CTC were transferred to glass slides, which were fixed with 4% PFA for 5 min. Wash the membrane by BD wash buffer (BD, USA) for three times. Then, add 100 ul Cytofix/ Permeabilization Kit (BD, USA) on the membrane for 20 min in order to allow for intracellular staining. After that, add 10% Goat serum to block for one hour. Then, discard the serum and add the primary mouse antibody to FITC-CK (1:100; Abcam, USA), rat antibody to PE-Vimentin (1:100; Abcam, USA) and rat antibody to AF647-CD45 (1:100; Santa, USA) for incubation overnight at 4 °C. On the next day, wash the membrane by BD wash buffer and add the secondary Alexa Fluor 488-conjugated goat anti-mouse IgG (1:100; Invitrogen, USA), Alexa Fluor 546-conjugated goat anti-rat IgG (1:200; Invitrogen, USA) and Alexa Fluor 647-conjugated goat anti-rat IgG (1:200; Invitrogen, USA). Nuclei was stained with Hoechst 33342 (1:500; Sigma, USA) and incubated for one hour, then wash the membrane three times with BD wash buffer. Finally, we imaged and enumerated CTC using a fluorescence microscopy (IX81; Olympus, Tokyo, Japan). CTC captured on membranes were photographed using IPP software (Media Cybernetics Inc., Silver Spring, MD, USA). CK+/Vimentin−/CD45−/Hoechst+ cell, CK−/Vimentin+/CD45−/Hoechst+ cell and CK−/Vimentin−/CD45−/Hoechst+ cell was defined as epithelial CTC (^E^CTC), mesenchymal CTC (^M^CTC) and white blood cell (WBC), respectively. In this study, ^M^CTC ratio refered to the ratio of the number of ^M^CTC to the total number of CTCs in 2.5 ml of peripheral blood per patient.

### Cell culture and reagents

The human monocyte cell line THP-1, HEK 293 T cells, human normal colon epithelial cell line NCM460 and CRC cell lines (HCT116, DLD-1, HT29, SW480, SW620 and Lovo) were purchased from the Chinese Academy of Sciences in Shanghai. Cells were cultured in RPMI 1640 medium (Gibco, ﻿USA) with 10% fetal bovine serum (FBS) (Gibco, ﻿USA) at 37 °C in a humidified atmosphere with 5% CO_2_. For macrophage generation, 3 × 10^5^ THP-1 cells were seeded in 0.4 μm sized pores inserts treated with 200 nM PMA (Sigma-Aldrich, ﻿USA) for 24 h and polarized into macrophages. To obtain TAMs, THP-1 macrophages were cultured by the addition of conditioned media from CRC cell lines (HCT116 or HT-29) for another 24 h. Morphologies of treated macrophages were observed and photographed under an inverted microscope (ZEISS, German). Macrophages and CRC cell lines co-cultivation was conducted using the non-contact co-culture transwell system (Corning, USA). Inserts containing TAMs or THP-1 macrophages were transferred to 6-well plate seeded with CRC cells (1 × 10^5^ cells per well) in advance and co-cultured. After 48 h of co-culture, TAMs or CRC cells were harvested for further analyses.

Recombinant human IL6 (R&D Systems) was dissolved in PBS containing 0.1% BSA and used at a final concentration of 50 ng/ml. STATTIC (STAT3 inhibitors), an anti-human neutralizing IL-6 antibody and an anti-human neutralizing CCL2 antibody were purchased from Med Chem Express, China.

### Plasmid constructs, siRNAs, miRNAs, and transfections

The STAT3 eukaryotic expression vector (NM_003150) and FoxQ1 ﻿plasmid vector (NM_033260) were chemically synthesized, constructed, sequenced and identified by Shanghai GeneChem Chemical Technology, Co. Ltd., China. Vectors of STAT3-siRNA (NM_003150), FoxQ1-siRNA (NM_033260), IL-6-siRNA (NM_000600) or negative control RNA (si-control) were also chemically synthesized, constructed, sequenced and identified by Shanghai GeneChem Chemical Technology, Co. Ltd., China. CRC cells (HCT116, HT-29) and TAMs were transfected with siRNAs or or negative control RNA using X-treme GENE siRNA Transfection Reagent (Roche, USA) according to the manufacturer’s instructions. Forty-eight hours after transfection, cells were plated for a functional assay or harvested for RNA and protein analyses. miR-506-3p mimics and inhibitor were obtained from RiboBio Co. Ltd., China. The RNA was transfected using Lipofectamine 2000 (Invitrogen, USA), following the manufacturer’s instructions. Stably transfected HCT116 and HT-29 cells were derived from the parental cells by puromycin (Sigma-Aldrich, USA) selection.

### Quantification of cytokines by enzyme-linked immunosorbent assay (ELISA)

The concentrations of cytokines were estimated for each experimental condition by ELISA, using commercial kits purchased from R&D Systems (Minneapolis, MN, USA), according to the manufacturer’s instructions. The cytokine kits included IL-10 (DY217B), IL-12 (DY1240), IL-1β (DY201), TNF-α (DY210), IFN-γ (DY285), and IL-6 (DY206). Positive controls were supplied in the kit.

### Flow cytometry

Macrophages were processed into single cell suspensions, incubated with antibodies (PE Mouse anti-Human CD163, APC Mouse anti-Human CD206, FITC Mouse anti-Human HLA-DR, APC-Cy7 Mouse anti-Human CD80, all from BD Biosciences, USA) for 1 h at 4 °C. The cells were then washed twice with 4 ml of flow buffer, then centrifuged, and resuspended in 0.5 ml of flow buffer for analysis. Flow cytometry was performed using a FACSCalibur flow cytometer (BD Biosciences, USA). Flow cytometric analysis was performed on FlowJo software (FlowJo, USA).

### RNA isolation and quantitative real-time PCR (qRT-PCR)

The total RNA from CRC cells and primary CRC xenograft tumor cells was isolated using the Trizol Reagent (Invitrogen, USA) according to the manufacturer’s instructions. After detection of RNA concentration, 1 μg of total RNA was reverse transcribed into cDNA using the PrimeScript™ RT reagent kit (Toyobo, Osaka). cDNA was used for subsequent qRT-PCR using the SYBR-Green PCR Master Mix (Takara, Osaka). Each reaction was run on the BioRad IQ5 Real time PCR machine (BioRad, USA). Relative expression was calculated using the 2^-ΔΔCt^ method. The sequences of primers used in the study are shown in Additional file 1: Table S3.

### Luciferase reporter assay

For miRNA target report assays, the 3′-UTR sequences of FoxQ1, and miRNA binding sites were amplified from the genomic DNA and sub-cloned into the psi-CHECK2 (Promega, USA). For the FoxQ1 promoter assay, a 2000-bp DNA fragment containing STAT3 binding sites upstream from the FoxQ1 promoter was cloned into pGL3-Basic plasmid (Promega, USA). For the miRNA promoter assay, miR-506-3p promoter (− 2000/+ 1) and its truncation (− 1753/+ 1, − 1298/+ 1, − 1137/+ 1 and − 856/+ 1) were amplified from genomic DNA by PCR, and inserted into pGL3-Basic (Promega, USA). The mutant constructs of STAT3 binding sites in the miR-506-3p promoter were generated using the QuikChange II Site-Directed Mutagenesis Kit (Stratagene, USA) and also cloned into pGL3-Basic vector. Cells (5 × 10^4^/well) were seeded at about 70% confluence in 24-well plates. For the miRNA target reporter assay, HEK293T were co-transfected with psi-CHECK-2 vectors and miRNA mimics, miRNA inhibitor or negative control using Lipofectamine 2000. For the STAT3-mediated miR-506-3p expression, pGL3-Basic luciferase reporters were transfected into HCT116 and HT29 cells after treated with IL-6 using Lipofectamine 2000. Renilla luciferase reporter vector pRL-SV40 (Promega, USA) was provided as an internal transfection control. The total cell lysates were harvested 48 h after transfection, and luciferase activities were determined using Dual-Luciferase reporter system (Promega, USA) according to the manufacturer’s instructions.

### Western blot

Cells were lysed using a RIPA buffer, including a protease inhibitor cocktail (Thermo Scientific, USA). The proteins were separated by SDS-PAGE gels and transferred to PVDF membranes (Millipore, USA). After blocking with 5% non-fat milk, the membranes were incubated with primary antibodies at 4 °C overnight. The HRP-conjugated secondary antibodies were used to incubate the membranes for 2 h at room temperature. The membranes were washed and incubated for 1 h at room temperature with HRP-conjugated secondary antibodies. Proteins were detected using a Bio-Rad ChemiDoc XRS + System. Bio-Rad Image Lab software was used for densitometric analysis. The following primary antibodies were purchased: anti-E-cadherin (1:1000; Cell Signaling, USA), anti-Vimentin (1:1000; Proteintech, USA), anti-p-JAK2 (1:1000; phosphor Y1007 + Y1008) (1:1000; Abcam, USA), anti-JAK2 (1:1000; Abcam, USA), anti-p-STAT3 (phosphor Y705) (1:1000; Cell Signaling, USA), anti-STAT3 (1:1000; Cell Signaling, USA), anti-p-AKT (phosphor S473) (1:1000; Abcam, USA), anti-AKT (1:1000; Abcam, USA), anti-p-ERK1/2 (phosphor T202 + T204) (1:1000; Cell Signaling, USA), anti-ERK1/2 (1:1000; Cell Signaling, USA), anti-FoxQ1 (1:1000; Sigma-Aldrich, USA), anti-GAPDH (1:5000; Santa Cruz, CA), anti-β-actin (Santa Cruz, CA).

### Colony formation and wound healing assay

For colony formation detection, 500 cells were planted in 6-well plates and cultured for 2 weeks. Cells were then fixed with 4% paraformaldehyde and stained with 0.5% crystal violet. The assay was performed three times for each treatment. A wound-healing assay was used to evaluate the ability of CRC cells to migrate following culture with TAMs. Cells were grown to 80–90% confluence in 24-well plates, and a wound was made by dragging a plastic pipette tip across the cell surface. The remaining cells were washed three times in PBS to remove cellular debris and incubated at 37 °C with serum-free medium. Migrating cells at the wound front were photographed after 24 h. All experiments were performed in triplicate. The area of the wound was measured with Image J software (NIH, USA).

### Transwell migration and invasion assay

Cell migration assays were performed using 24-well Transwells (8 μm pore size; Corning, USA) uncoated with Matrigel. Cell invasion assays were performed using 24-well Transwells (8 μm pore size; Corning, USA) pre-coated with Matrigel (Falcon 354,480; BD Biosciences, USA). In total, 1 × 10^5^ cells were suspended in 500 μl RPMI 1640 containing 1% FBS and added to the upper chamber, while 750 μl RPMI 1640 containing 10% FBS was placed in the lower chamber. After 48 h of incubation, Matrigel and the cells remaining in the upper chamber were removed using cotton swabs. Cells on the lower surface of the membrane were fixed in 4% paraformaldehyde and stained with 0.5% crystal violet. Cells in 5 microscopic fields (at × 200 magnification) were counted and photographed. All experiments were performed in triplicate.

### Chromatin immunoprecipitation (ChIP) assay

ChIP assays were performed using a SimpleChIP^®^ Enzymatic Chromatin IP Kit (Cell Signaling, #9003, USA) according to the manufacturer’s instructions. The resulting precipitated DNA specimens were analyzed by using PCR to amplify a 106-bp region (CHIP 1) of the miR-506-3p promoter with the primers 5′-ACC CAT GAA ATC ATC CCC TA-3′ (forward) and 5′-TGT GCA GAA GAC CGA AAA TG-3′ (reverse) and a 146-bp region (CHIP 2) of the miR-506-3p promoter with the primers 5′-TGT GTG TAT GAG CAT GTG TTT G-3′ (forward) and 5′-GAT TTA GGG GAT GAT TTC ATG G-3′ (reverse). The negative control is an encoding region of miR-506-3p, which was amplified by PCR with the primers 5′-GTG CCA TTT TAC TTT CCT ACC-3′ (forward) and 5′-TAG GGA AAT GCA ACC AAA ACC-3′ (reverse). The PCR products were resolved electrophoretically on a 1% agarose gel and visualized with use of ethidium bromide staining.

### Animal experiments

All animal experiments were performed according to our institutions’ guidelines for the use of laboratory animals and were approved by the Institutional Animal Care and ethical committee of Zhongnan hospital of Wuhan University. For the tumor growth assay, the 6–8 weeks old nude mice were divided into four randomized groups (*n* = 6 per group), and HCT116 cells alone (5 × 10^5^), TAMs alone (5 × 10^5^), HCT116 cells (5 × 10^5^) and TAMs/si-control (5 × 10^5^), or HCT116 cells (5 × 10^5^) and TAMs/si-IL-6(5 × 10^5^) ﻿in 200 μl were subcutaneously injected into the flank of each mouse. After 10 days, we began measuring the tumor size every 5 days using digital vernier calipers, and calculated the tumor volume according to the following formula: volume = 1/2 × (width^2^ × length). Thirty days after cell injection, 1 ml of blood was collected via cardiac puncture into EDTA-containing tubes (BD, USA), the mice were sacrificed to collected the tumors and visually examined. For the liver and lung metastasis experiment, the 6–8 weeks old nude mice were divided into three randomized groups (*n* = 6 per group), and HCT116 cells alone (5 × 10^5^), HCT116 cells (5 × 10^5^) and TAMs/si-control (5 × 10^5^), or HCT116 cells (5 × 10^5^) and TAMs/si-IL-6(5 × 10^5^) ﻿in 100 μl were injected into the mice via tail vein. Thirty days after cell injection, the mice were euthanized and were necropsied to assess metastatic burden. The tumor tissues, liver and lung tissues of mice were further examined by H&E, IHC staining, or RT-PCR assay.

### Statistics analysis

All statistical analyses were performed with SPSS statistical software (version 22.0, IBM SPSS, USA) and GraphPad Prism software (version 6.0, GraphPad Software, USA) for Windows. Pearson’s correlation analysis was performed to assess the relationship between CD68, CD163 expression and ^M^CTC ratio in the PB of patients. Chi-square test was applied to analyze the relationship between CD68 and CD163 expression and clinicopathological status. Groups of discrete variables were compared by means of the Mann-Whitney *U* test or Kruskal-Wallis nonparametric analysis of variance. Kaplan–Meier method was used for survival analysis and drawing the survival curves, and difference among patients’ subgroups was calculated by log-rank test. Univariate and multivariate Cox-regression analyses were applied to identify the independent factors of prognosis. All experiments for cell cultures were performed independently at least three times and in triplicate each time. In all cases, *P* values < 0.05 were considered statistically significant.

## Results

### CD163^+^ TAMs at invasive front is correlated with EMT, ^M^CTC ratio, and poor prognosis in CRC patients

**Fig. 1:**
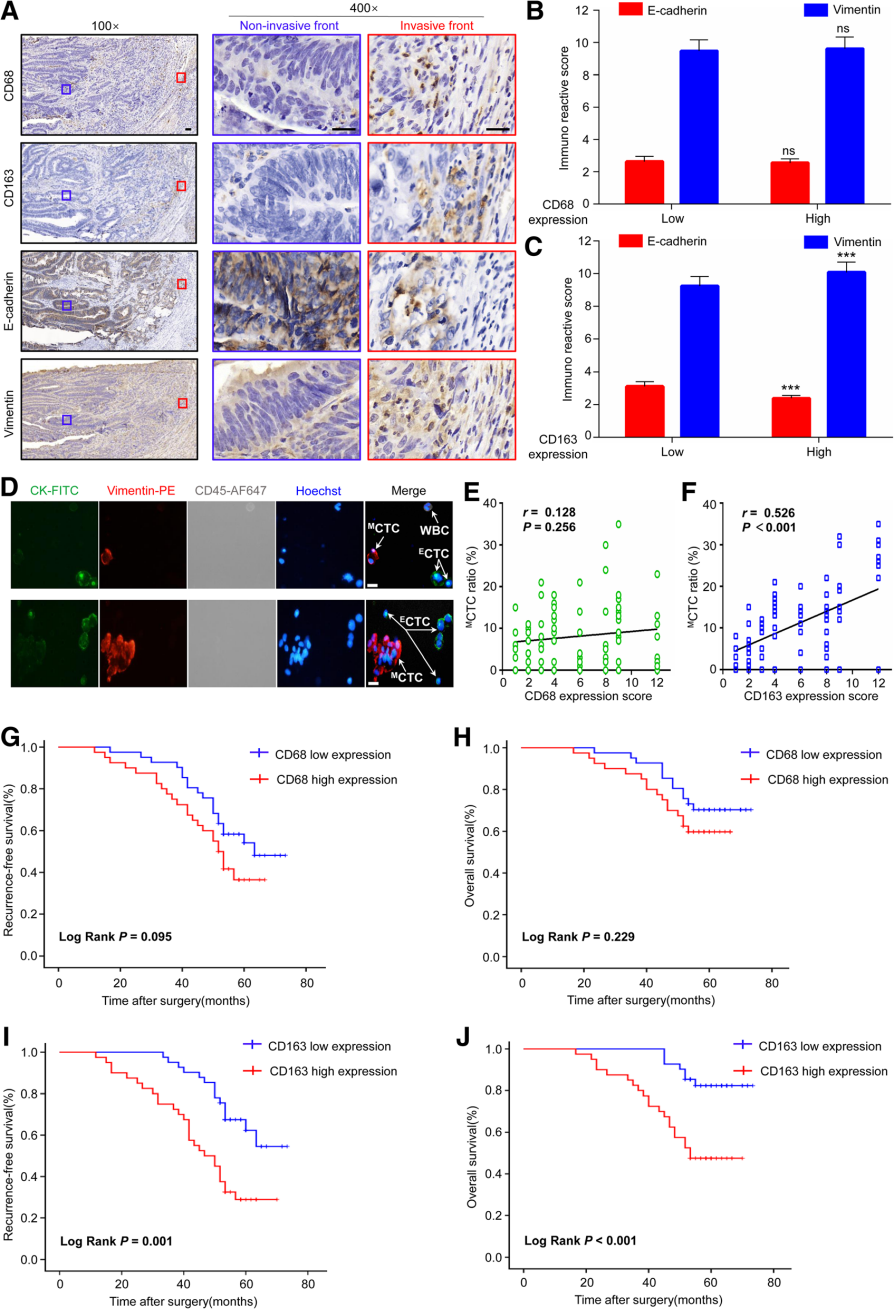
CD163^+^ TAMs at invasive front is correlated with EMT phenotype, ^M^CTC ratio, and poor prognosis in CRC patients. (**a**) Representative IHC staining for CD68, CD163, E-cadherin, and Vimentin in the invasive front and non-invasive front of serial sections from a human CRC sample. (**b**-**c**) Expression of E-cadherin and Vimentin in human CRC samples with low or high CD68 and CD163 expression at invasive front, respectively. (**d**) Representative CTC images from included patient 5 and 27, respectively. Four-color immunocytochemistry method based on FITC-labeled anti-CK, PE-labeled anti-Vimentin, AF647-labeled anti-CD45, and Hoechst nuclear staining was applied to identify and enumerate CTCs from non-specially trapped WBCs. Scale bar, 20 μm. (**e**-**f**) Association of CD68 and CD163 expression at invasive front witth ^M^CTC ratio, respectively. (**g**-**h**) Association of CD68 expression at invasive front with the patients’ recurrence-free survival and overall survival in CRC, respectively. (**i**-**j**) Association of CD163 expression at invasive front with the patients’ recurrence-free survival and overall survival in CRC, respectively. Error bars, SEM. ns, not significant; ***P < 0.001

To determine the clinical significance of TAMs in CRC, we firstly examined the expression of TAMs markers (CD68, CD163) and EMT markers (E-cadherin, Vimentin) in serial sections from 81 CRC cases. Intriguingly, we found that CD68 and CD163 were mainly expressed at the tumor invasive front and stroma, with no to weak expression in tumor nest (Fig. 1A). Furthermore, near tumor invasive front, high level of CD163 was associated with less E-cadherin and more Vimentin, an indication of EMT (Fig. 1A-C). At the same time, the level of CD68 was not associated with the EMT program (Fig. 1A-C). However, at tumor stroma neither CD163 nor CD68 expression was associated with the EMT program (Additional file 1: Figure S1A and S1B).

We further explored the relationship between the expression of CD163 and CD68 with ^M^CTC ratio from 2.5 ml peripheral blood of included patients. The representative images of CTC from patient 5 and 27 were presented in Fig. 1D. Overall, the ^M^CTC ratio was ranged from 0 to 35%, with an average percentage of 11.09 ± 9.16%. Interestingly, further analyses found that ^M^CTC ratio was significantly associated with the expression of CD163 (r = 0.526, *P* < 0.001) (Fig. 1F), but not with CD68 at tumor invasive front (r = 0.128, *P* = 0.256) (Fig. 1E). By contrast, either CD163 or CD68 expression at tumor stroma was insignificantly associated with ^M^CTC ratio (Additional file 1: Figure S1C and S1D).

Next, we investigated the correlations of CD163 and CD68 expression with clinicopathological parameters of CRC patients. As shown in Table 1, high expression of CD163 at tumor invasive front was significantly associated with tumor grade, LVI, TI, LNM and TNM stage (*P* < 0.05, respectively), while high expression of CD68 at tumor invasive front was only significantly associated with LNM (*P* = 0.016). In contrast to the strong clinical associations of the tumor invasive front populations, only tumor grade was found to be correlated with the high expression of CD163 at tumor non-invasive front (*P* < 0.05), while none of clinicopathological factors was found to be correlated with the expression of CD68 at tumor non-invasive front in this study (*P* > 0.05 for all) (Additional file 1: Table S1). Further prognostic analysis revealed that, at the invasive front of CRC, high level of CD68 expression was insignificantly associated with worse RFS (*P* = 0.095) (Fig. 1G) and OS (*P* = 0.229) (Fig. 1H), however, high level of CD163 expression was significantly correlated with poor RFS (*P* = 0.001) (Fig. 1I) and OS (*P* < 0.001) (Fig. 1J). Either CD68 or CD163 expression at non-invasive front was not associated with the prognosis of CRC patients (Additional file 1: Figure S1E-S1H). Univariate and multivariate analyses showed that CD163 expression at the invasive front was an independent prognostic factor associated with poor RFS (HR = 2.414, 95%CI = 1.016–4.523, *P* = 0.045) and OS (HR = 3.234, 95%CI = 1.176–8.889, *P* = 0.023) (Table 2). These data indicate that CD163^+^ TAMs at invasive front promote the release of ^M^CTC by mediating the EMT program of primary tumor cells, thereby promoting tumor progression and affecting the prognosis of CRC patients.

**Table 1:** Correlation between the density of macrophages at invasive front and clinicopathologic parameters (*n* = 81)

Parameters	n (%)	CD68 expression			CD163 expression		
		Low	High	*P*	Low	High	*P*
Gender				0.855			0.893
Male	48 (59.3)	25	23		24	24	
Female	33 (40.7)	16	17		17	16	
Age, years				0.908			0.575
<60	39 (48.1)	20	19		21	18	
≥60	42 (51.9)	21	21		20	22	
Tumor site				0.908			0.742
Colon	42(51.9)	21	21		22	20	
Rectal	39 (48.1)	20	19		19	20	
Tumor size, cm				0.271			
<5	54 (66.7)	25	29		28	26	0.753
≥5	27 (33.3)	16	11		13	14	
Tumor grade				0.715			**0.018**
Poor	32 (39.5)	17	15		11	21	
Moderate/Well	49(60.5)	24	25		30	19	
LVI				0.734			**0.005**
Absence	45(55.6)	23	22		29	16	
Presence	36(44.4)	18	18		12	24	
PNI				0.582			0.320
Absence	43(53.1)	23	20		24	19	
Presence	38(46.9)	18	20		17	21	
TI				0.228			**0.014**
T1–2	12(14.8)	8	4		10	2	
T3–4	69(85.2)	33	36		31	38	
LNM				**0.016**			**< 0.001**
N0–1	53(65.4)	32	21		36	17	
N2–3	28(34.6)	9	19		5	23	
TNM stage^a^				0.096			**0.011**
I/II	42(51.9)	25	17		27	15	
III	39(48.1)	16	23		14	25	
CA19–9, U/mL				0.296			0.576
<37	57(70.4)	31	26		30	27	
≥37	24(29.6)	10	14		11	13	
CEA, ng/ml				0.689			0.939
<5	55(67.9)	27	28		28	27	
≥5	26(32.1)	14	12		13	13	
Overall	81(100.0)	41	40		41	40	

Notes: ^a^The 8th edition of the AJCC Cancer Staging Manual; Boldface indicates *P* < 0.05

*Abbreviations:* *LVI* lymphovascular invasion, *PNI* perineural invasion, *TI* tumor invasion, *LNM* lymph node metastasis, *TNM* tumor-node-metastasis, *CA19–9* carbohydrate antigen 19–9, *CEA* carcinoembryonic antigen, *CD68* cluster of differentiation 68, *CD163* cluster of differentiation 163

**Table 2:** Univariate and multivariate analyses of clinicopathologic parameters associated with recurrence-free survival and overall survival

Parameters	Recurrence-free survival						Overall survival					
	Univariate analysis			Multivariate analysis			Univariate analysis			Multivariate analysis		
	HR	95% CI	*P*	HR	95% CI	*P*	HR	95% CI	*P*	HR	95% CI	*P*
Gender (Female vs Male)	0.960	0.517–1.783	0.897				1.025	0.480–2.189	0.949			
Age (<60 years vs ≥60 years)	1.168	0.642–2.126	0.612				1.579	0.739–3.372	0.238			
Tumor site (Colon vs Rectal)	0.753	0.412–1.375	0.355				0.644	0.302–1.376	0.256			
Tumor size (<5 cm vs ≥ 5 cm)	0.816	0.431–1.546	0.534				0.586	0.249–1.381	0.222			
Tumor grade (Well & Moderate vs Poor)	0.627	0.424–0.927	**0.019**	0.883	0.593–1.315	0.540	0.586	0.354–0.967	**0.037**	0.757	0.453–1.266	0.289
LVI (Presence vs Absence)	2.756	1.477–5.143	**0.001**	1.495	0.757–2.954	0.247	2.570	1.185–5.572	**0.017**	1.284	0.540–3.051	0.572
PNI (Presence vs Absence)	1.893	1.032–3.471	**0.039**	2.193	1.136–4.233	**0.019**	2.441	1.125–5.294	**0.024**	2.981	1.262–7.039	**0.013**
TI (T3–4 vs T1–2)	1.977	1.228–3.182	**0.005**	1.569	0.833–2.955	0.163	2.634	1.375–5.049	**0.004**	1.759	0.778–3.977	0.175
LNM (N2–3 vs N0–1)	1.967	1.406–2.753	**< 0.001**	1.155	0.627–2.127	0.644	2.084	1.380–3.147	**< 0.001**	0.891	0.410–1.935	0.770
TNM stage^a^ (III vs II vs I)	4.070	2.172–7.629	**< 0.001**	2.409	0.793–7.317	0.121	8.401	2.964–23.811	**< 0.001**	7.113	1.505–33.627	**0.013**
CA19–9 (≥37 U/ml vs < 37 U/mL)	1.609	0.866–2.988	0.132				1.214	0.549–2.684	0.631			
CEA (≥5 ng/ml vs < 5 ng/ml)	1.331	0.716–2.471	0.366				1.464	0.685–3.126	0.325			
Tumor invasive front (High vs Low)												
CD68	1.653	0.091–3.033	0.105				1.571	0.743–3.322	0.237			
CD163	2.861	1.523–5.376	**0.001**	2.144	1.016–4.523	**0.045**	4.149	1.761–9.776	**0.001**	3.234	1.176–8.889	**0.023**
Non-tumor invasive front (High vs Low)												
CD68	1.515	0.825–2.781	0.180				1.364	0.645–2.884	0.416			
CD163	1.231	0.676–2.242	0.498				1.916	0.884–4.152	0.100			

Notes: ^a^The 8th edition of the AJCC Cancer Staging Manual; Boldface indicates *P* < 0.05

Abbreviations: *LVI* lymphovascular invasion, *PNI* perineural invasion, *TI* tumor invasion, *LNM* lymph node metastasis, *TNM* tumor-node-metastasis, *CA19–9* carbohydrate antigen 19–9, *CEA* carcinoembryonic antigen, *CD68* cluster of differentiation 68, *CD163* cluster of differentiation 163

### CD163^+^ TAMs induce EMT to promote migration and invasion of CRC cells

**Fig. 2:**
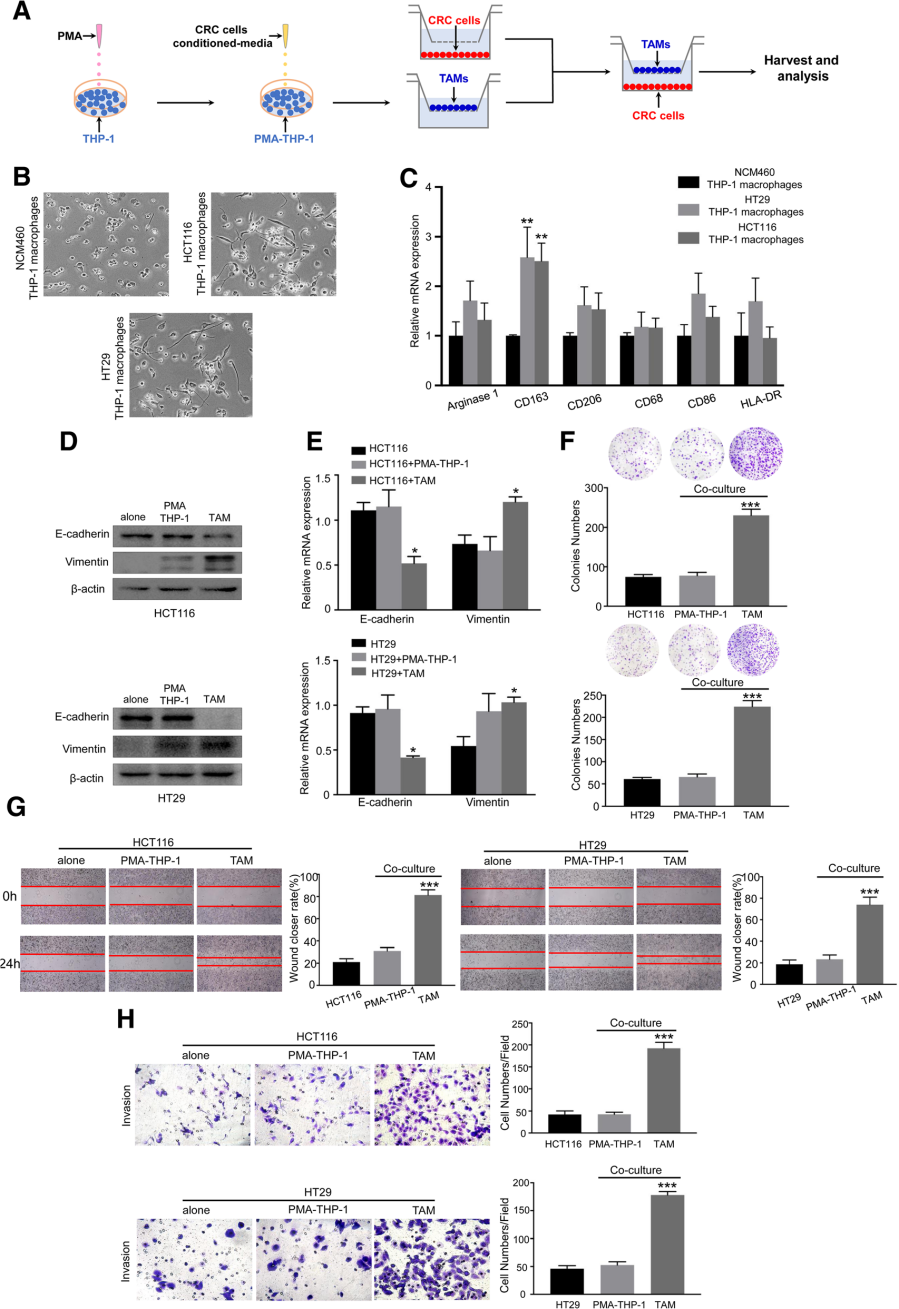
CD163^+^ TAMs induce EMT to promote migration and invasion of CRC cells. (**a**) Schema for representing the experiment procedures. (**b**) PMA-treated THP-1 macrophages were cultured with NCM460-, HCT116- or HT29-conditioned media for 48 h. The representative bright-field images of macrophages treated by the respective conditioned media are shown. (magnification, ×200). (**c**) RT-PCR analyzed the expression of the markers of pan-macrophage (CD68), M1 (arginase 1, CD86, HLA-DR) and M2 (CD163, CD206) macrophages in PMA-treated THP-1 macrophages incubated with the conditioned media from NCM460, HCT116 and HT29 for 48 h; Error bars, SEM. (**d**) The effect of the TAMs on the EMT of CRC cells (HCT116 and HT29) was analyzed by Western blot analysis. (**e**) RT-PCR for analyzing the expression of E-cadherin and Vimentin in CRC cells (HCT116 and HT29) alone or co-cultured with macrophages (PMA-treated THP-1 macrophages or TAMs) for 48 h; Error bars, SEM. (**f**), (**g**) and (**h**) Cell proliferation, migration and invasion capacity of CRC cells (HCT116 and HT29) alone or co-cultured with macrophages (PMA-treated THP-1 macrophages or TAMs) was determined by the colony formation, wound healing assay and transwell coculture system, respectively. Representative photographs of migratory or invaded cells (magnification, × 200) are shown; Error bars, SD. **P < 0.01; ***P < 0.001

To determine the above clinical results, we utilized an in vitro model of tumor-associated macrophages. The human monocyte cell line THP-1 was induced into macrophages by treatment with PMA for 24 h, and then cultured with conditioned media (CM) from different CRC cell lines (HCT116 or HT29) to generate TAMs (Fig. 2A), which were validated on the basis of morphology, marker expression, and cytokine profile. Macrophages treated with CM from HT-29 or HCT116, but not normal cell line (NCM460), became stretched and elongated (Fig. 2B) and exhibited higher levels of M2 marker CD163 but not mannose receptor CD206 (Fig. 2C). Flow cytometry validated the increased CD163 in HT-29 or HCT116 conditioned macrophages compared with NCM460 (Additional file 1: Figure S2A). HT-29 or HCT116 conditioned macrophages expressed higher levels of the “alternatively-activated M2” marker IL-10, but not the “classically-activated M1” marker IL-12 (Additional file 1: Figure S2B). Interestingly, HT-29 or HCT116 conditioned macrophages also showed strong expression of the pro-inflammatory cytokines, including IL-1β, IFN-γ, and TNF-α similar to the in vitro polarized M1-macrophages (Additional file 1: Figure S2C). Together, these data indicate that tumor cells induced TAMs of a mixed M1/M2 phenotype.

To investigate whether TAMs could induce EMT of CRC cells in vitro, Western blot and RT-PCR were performed to analyze the EMT markers in HT-29 or HCT116 cells after being co-cultured with TAMs in a non-contact Transwell system that allowed the exchange of soluble factors, but were impermeable for cells themselves (Fig. 2A). As shown in Fig. 2D and E, the expression of epithelial marker E-cadherin was reduced, while the mesenchymal marker Vimentin was up-regulation. Compared with the control, TAMs co-culture led to a spindle-shaped morphology, loss of cell-to-cell contact, and increased formation of pseudopodia in HCT116 and HT29 cells (Additional file 1: Figure S2D). Meanwhile, to further verify whether TAMs directly induced the growth of CRC cells, after 48 h of TAMs co-culture, HT-29 and HCT116 cells were subjected to clonogenic assay. As shown in Fig. 2F, TAMs co-culture significantly increased the clonogenic survivals compared with control. Wound-healing assay and transwell assay were used to determine whether TAMs could promote the migratory and invasive abilities of HT-29 or HCT116. Compared with control, TAMs co-culture exhibited a faster closure of the wound. The result was confirmed by transwell assay (Fig. 2G and H). Taken together, our findings show TAMs mediated EMT to promote proliferative, migratory and invasive behaviors in CRC cells.

### IL6 is required for TAMs-induced EMT of CRC cells

**Fig. 3:**
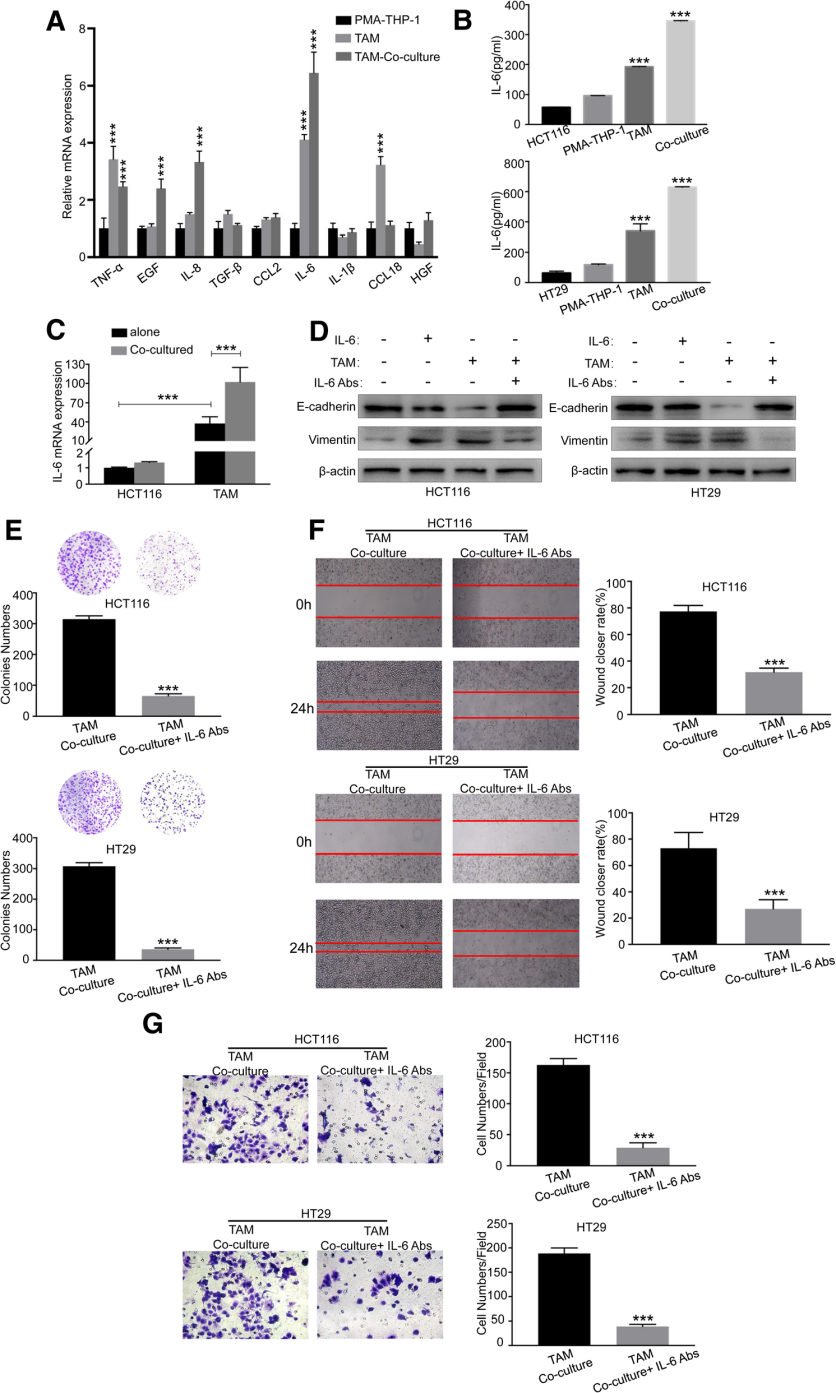
IL6 is required for TAMs-induced EMT of CRC cells. (**a**) Relative expression levels of representative EMT/inflammation related-cytokines were detected in TAMs co-cultured with HCT116 as determined by RT-PCR; Error bars, SEM. (**b**) ELISA assay of IL6 protein secretion of CRC cells (HCT116 and HT29) and various macrophages; Error bars, SD. (**c**) IL6 mRNA expression in HCT116 and TAMs with or without 48 h of coculture. Error bars, SEM. (**d**) Expression of EMT markers E-cadherin and Vimentin in CRC cells (HCT116 and HT29) alone, IL6-supplemented CRC cells, TAMs-co-cultured CRC cells, and IL6 depleted TAMs-co-cultured CRC cells were analyzed by Western blot. (**e**) Colony formation assay was used to quantify the number of spheres of IL6 depleted TAMs-co-cultured CRC cells (HCT116 and HT29) and its control; Error bars, SD. (**f**) Migration of IL6 depleted TAMs-co-cultured CRC cells (HCT116 and HT29) and its control was measured by wound-healing assay. Error bars, SD. (**g**) Invasion of IL6 depleted TAMs-co-cultured CRC cells (HCT116 and HT29) and its control was measured by transwell (magnification, × 200); Error bars, SD. ***P < 0.001

Given that cytokine secretion represents the major functional response of macrophages, it was speculated that a signaling mechanism between TAMs and CRC cells exists that accounts at least in part for the previously described pro-tumorigenic activities [27]. To identify the TAMs-derived factors, we conducted a RT-PCR analysis of 9 cytokines related to the inflammation/ EMT axis, and found that the mRNA levels of IL6 emerged as the most prominently upregulated and abundant cytokine in co-cultured TAMs with HCT116 cells than those in THP-1 macrophages or TAMs cultured alone (Fig. 3A). ELISA further showed that IL6 levels were significantly increased in the media from co-cultured TAMs with HCT116 cells compared to those from THP-1 macrophages, TAMs, or HCT116 alone (Fig. 3B). In HT-29 cells, similar results were obtained (Fig. 3B). The basal level of IL6 mRNA was much higher in TAMs than HCT116 cells, and HCT116 co-cultured with TAMs promoted IL6 expression in TAMs but not in HCT116 cells (Fig. 3C). These results suggested that most of the IL6 was derived from TAMs, consistent with the results from ELISA (Fig. 3B). To evaluate whether IL6 was critical for the EMT in CRC, an exogenous recombinant IL6 was added in the culture medium of CRC cell lines. The results showed that IL6 significantly increased the expression of Vimentin, while reduced the expression of E-cadherin (Fig. 3D). Furthermore, an IL6 neutralizing antibody was used to confirm TAMs-induced EMT in CRC through IL6. After applied IL6 neutralizing antibody in TAM-co-culture medium, the expression of E-cadherin was increased while Vimentin was decreased (Fig. 3D). Besides, treatment of IL6 neutralizing antibody significantly inhibited the colony formation ability of CRC cells co-cultured with TAMs (Fig. 3E). Consistently, the depletion of IL6 decreased migratory (Fig. 3F) and invasive capacities (Fig. 3G) of CRC cells in vitro. These results demonstrate that TAMs-derived IL6 is one of the major cytokines that may mediate the interplay between TAMs and CRC cells.

### IL6/STAT3/FoxQ1 contributes to TAMs-induced EMT and macrophages attraction

**Fig. 4:**
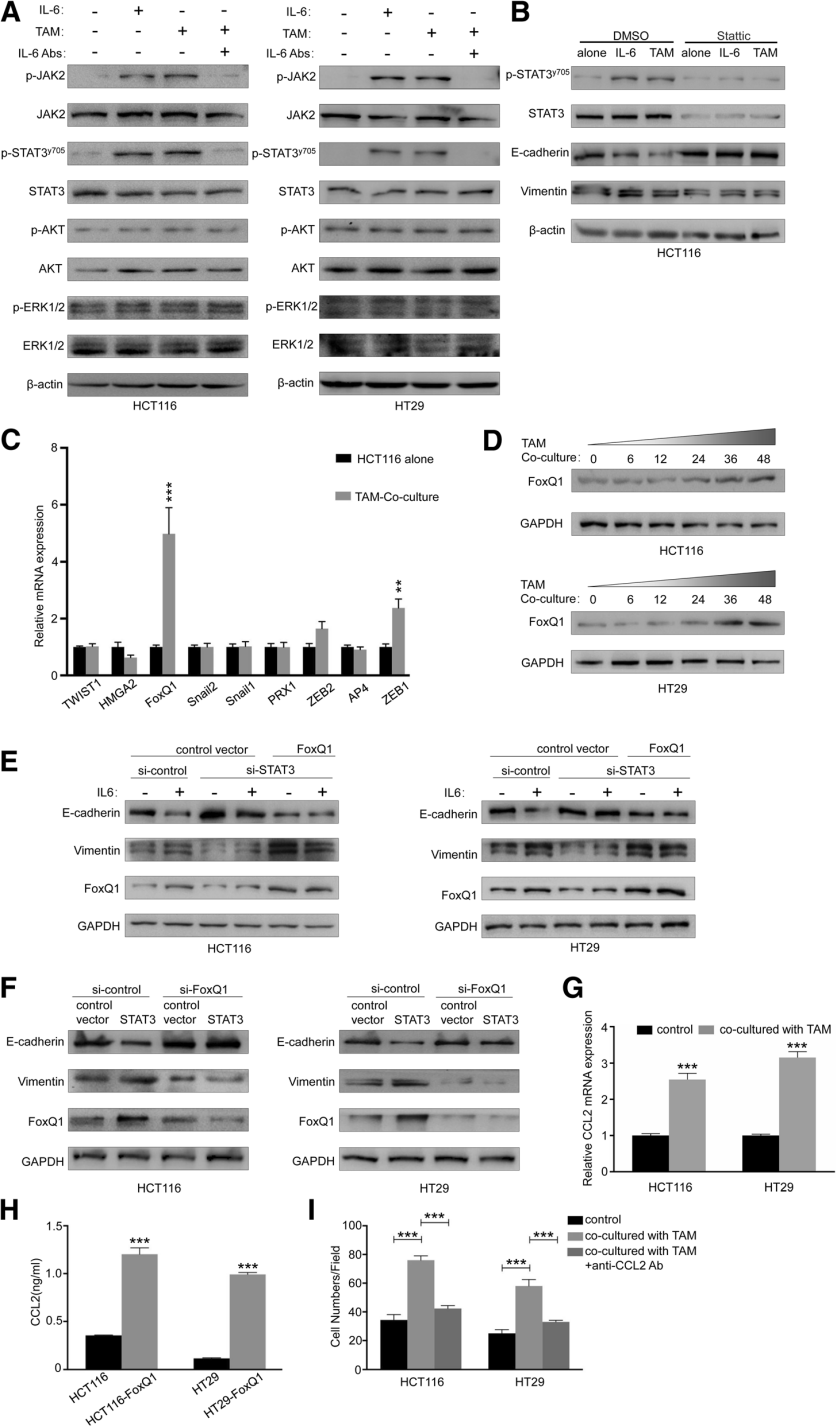
STAT3/FoxQ1 contributes to TAMs-induced EMT and macrophages attraction. (**a**) Western blot analysis of CRC cells (HCT116 and HT29) alone, IL6-supplemented CRC cells, TAMs-co-cultured CRC cells, and IL6 depleted TAMs-co-cultured CRC cells. (**b**) Western blot analysis of HCT116 cells alone, IL6-supplemented HCT116, TAMs-co-cultured HCT116 in the presence or absence of Stattic (15 ﻿μM). (**c**) Relative expression levels of representative EMT related factors were detected in HCT116 with or without 48 h of TAMs co-culture as determined by RT-PCR. Error bars, SEM. (**d**) Western blot of FoxQ1 from whole-cell lysates extracted from CRC cells (HCT116 and HT29) co-cultured with TAMs for the indicated times. (**e**) Western blot of colorectal cancer cells (HCT116 and HT29) transfected with FoxQ1 expression vector or empty vector and indicated siRNAs (si-STAT3) and incubated with IL6 for 48 h afterwards. (**f**) Western blot of CRC cells (HCT116 and HT29) transfected with STAT3 expression vector or empty vector and indicated siRNAs (si-FoxQ1). (**g**) CCL2 mRNA expression in CRC cells (HCT116 and HT29) with or without 48 h of TAMs co-culture; Error bars, SD. (**h**) ELISA assay of CCL2 protein secretion of CRC cells (HCT116 and HT29) transfected with FoxQ1 expression vector or empty vector. Error bars, SD. (**i**) ﻿THP-1 cells migration towards CRC cells (HCT116 and HT29) alone and TAMs-co-cultured CRC cells with or without anti-CCL2 Ab; Error bars, SD. ***P < 0.001

To determine what downstream signals in the tumor cells responded to IL6 secretion by TAMs, we looked at ERK, Akt, and STAT3 pathway, which all have been reported to be activated upon stimulation with IL6 [28, 29]. The results found that the stimulation of CRC cells with IL6 or co-cultured with TAMs increased the expressions of p-JAK2 and p-STAT3, whereas the treatment of IL6 neutralizing antibody inhibited co-cultured-induced expressions of p-JAK2 and p-STAT3 (Fig. 4A). To investigate the role of JAK2/STAT3 signaling in TAMs-induced EMT, treatment of Stattic, a STAT3 inhibitor, markedly blocked IL6 or co-cultured-induced expressions of p-STAT3, and also attenuated EMT in HCT116 (Fig. 4B). Consistently, similar attenuation was observed while knockdown of STAT3 (Fig. 4E). These data demonstrate that TAMs elicited EMT through activating JAK2/STAT3 signaling in CRC cells.

EMT is primarily controlled by transcription factors, which act to regulate the repression of epithelial marker proteins, and induce mesenchymal gene expression [10]. Based on the above findings that TAMs may regulate EMT, we next detected the expressions of Twist1, HMGA2, FoxQ1, Snail2, Snail1, PRX1, ZEB2, AP4, and ZEB1 by RT-PCR in HCT116 cells co-cultured with TAMs. Among them, FoxQ1 showed most significant up-regulation (Fig. 4C). Meanwhile, the protein levels of FoxQ1 were also increased upon co-cultured with TAMs in a time dependent manner (Fig. 4D). Given that the activation of STAT3 and upregulation of FoxQ1 were involved in TAMs-triggered EMT, we speculated that STAT3 activation was associate with upregulation of the FoxQ1 expression. In agreement with this idea, knockdown of STAT3 markedly attenuated the IL6 induced FoxQ1 expression (Fig. 4E). Meanwhile, STAT3 silencing had a destructive effect on the EMT process, while ectopic expression of FoxQ1 largely restored Vimentin and decreased E-cadherin expressions in CRC cells (Fig. 4E). By contrast, overexpression of STAT3 significantly increased FoxQ1 expression compared with the control (Fig. 4F). Moreover, overexpression of STAT3 significantly reduced E-cadherin and enhanced Vimentin expressions, while STAT3-induced EMT was significantly inhibited by knockdown of FoxQ1 expression (Fig. 4F). These results indicate that STAT3, participating in TAMs-induced EMT in CRC cells, depends on FoxQ1.

According to the above results that TAMs at invasive front mediate the EMT program of tumor cells, we speculated EMT-programmed tumor cells can in turn recruit macrophages. Tumor cells produce numerous chemokines that attract macrophages, which are capable of producing an assorted array of cytokines, such as IL6 as shown above, all of which dictate the fate of a developing tumor. It is well-established that CCL2 is a chemokine that is essential for the recruitment of macrophage cells [30], and FoxQ1 expressed in cancer cells has shown to increase attraction of macrophages through CCL2 production [31]. ﻿Accordingly, we examined the expression ﻿of CCL2 in human CRC cell lines by RT-PCR. ﻿HT-29 or HCT116 cells co-cultured with TAMs exhibited higher expression of CCL2 than tumor cells alone (Fig. 4G). We also examined CCL2 protein level in supernatants of the human CRC cell lines by ELISA, and found that up-regulation of FoxQ1 increased CCL2 secretion from HCT116 and HT29 cells (Fig. 4H). To investigate whether EMT-CRC cells induced by TAMs could attract macrophages infiltration into TME, the chemotaxis of THP-1 monocytes towards CRC cells was detected. Monocyte cells showed significant migration to HCT116 or HT29 cells after co-cultured with TAMs compared with control (Fig. 4I). To confirm that the enhanced migration of monocytes was mediated by CCL2, anti-CCL2-neutralizing Ab treatment significantly inhibited the TAMs-enhanced migration of monocytes (Fig. 4I). Collectively, these data suggest that a positive feedback loop between IL6 from TAMs and CCL2 from TAMs-educated CRC cells promotes the EMT of cancer cells and the recruitment of macrophages.

### STAT3 regulates FoxQ1 through miR-506-3p in a post-transcription manner

**Fig. 5:**
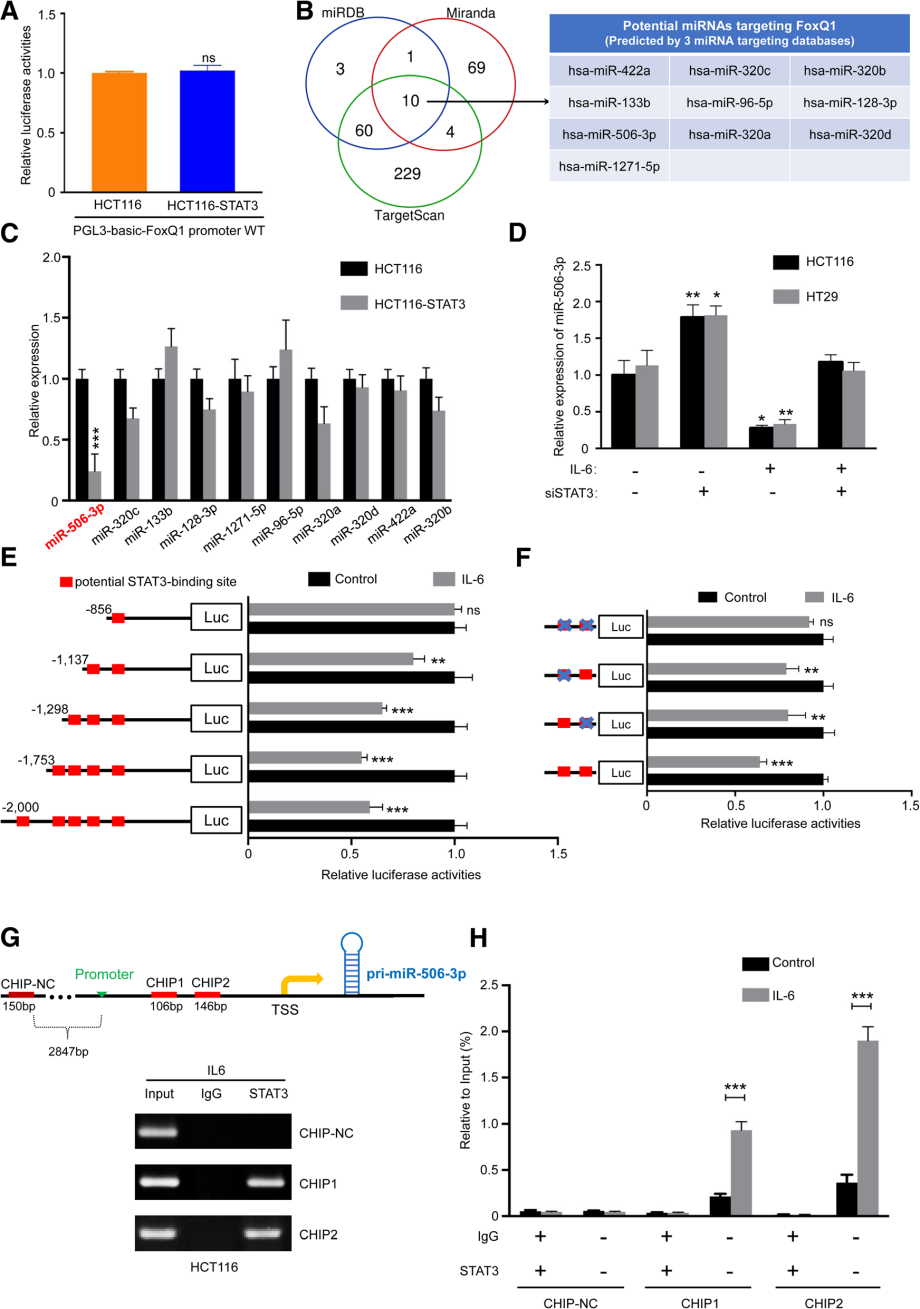
STAT3 activation downregulates miR-506-3p expression. (**a**) Overexpression of STAT3 didn’t cause an increase in FoxQ1 promoter luciferase activity in HCT116 cells. (**b**) Three independent miRNA target databases were used to predict the potential miRNAs. (**c**) Relative expression levels of representative nine potential miRNAs were detected in HCT116 transfected with STAT3 expression vector or empty vector as determined by RT-PCR. Error bars, SEM. (**d**) CRC cells (HCT116 and HT29) were infected with indicated siRNAs (si-STAT3) and treated with IL6 (50 ng/ml) or control for 48 h, and expression of miR-506-3p was examined using RT-PCR; Error bars, SEM. (**e**) Serially truncated and mutated miR-506-3p promoter constructs were cloned to pGL3-Basic luciferase reporters and transfected into HCT116 cells. The relative luciferase activities were determined after IL6 (50 ng/ml) treatment for 1 h; Error bars, SD. (**f**) Selective mutation analyses identified STAT3-responsive regions in the miR-506-3p promoter in HCT116 cells; Error bars, SD. (**g**) ChIP assay demonstrated the direct binding of STAT3 to the miR-506-3p promoter, including nonspecific control (N.C), CHIP1, and CHIP2 in HCT116 cells. Input, 5% of total lysate. (**h**) RT-PCR of the ChIP products confirmed the direct binding capacity of STAT3 to the miR-506-3p promoter in HCT116 cells. Input, 5% of total lysate; Error bars, SD. ns, not significant; *P < 0.05; **P < 0.01; ***P < 0.001

To investigate whether STAT3 directly activated FoxQ1 mRNA transcription, we cloned the promoter region of FoxQ1 with a luciferase reporter. However, the luciferase activity did not change despite of overexpression of STAT3 in HCT116 cells, which indicated that FoxQ1 expression might be regulated by STAT3 in a post-transcription manner (Fig. 5A). Next, we considered whether miRNAs targeting 3′ UTR of FoxQ1 could be regulated by STAT3, and used three independent databases (TargetScan, miRanda and miRDB) to predict miRNAs that may be involved in silico. Noticeably, 10 miRNAs were predicted by all three tools and we detected a repressed expression of miR-506-3p under overexpression of STAT3, but no other nine candidates in HCT116 cells (Fig. 5B and C). The RT-PCR analysis revealed that miR-506-3p expression decreased in HT-29 and HCT116 cells treated with IL6 (Fig. 5D). Moreover, knockdown of STAT3 prevented the repression effect of IL6 on miR-506-3p (Fig. 5E). To validate whether transcription of miR-506-3p was directly activated by STAT3, we performed bioinformatics analysis of miR-506-3p promoter region to reveal potential transcription factor binding sites and five potential STAT3-binding sites were revealed by Jaspar [32] (Additional file 1: Figure S3A and Additional file 1: Table S2). We generated a series of 5′ deletion constructs of miR-506-3p promoter and determined whether STAT3 transcriptionally suppressed miR-506-3p. A luciferase assay after IL6 treatment showed that the regulatory region between − 1298 and − 856 bp was responsible for STAT3-mediated promoter regulation, whereas the remaining modified miR-506-3p promoters failed to shut down STAT3-suppressed reporter system (Fig. 5E and Additional file 1: Figure S3B). Two STAT3-binding sites are located in this region. Reporter genes containing the miR-506-3p promoters with mutations in the potentially candidate binding sites were transfected into HCT116 and HT29 cells, and then, these cells were treated with IL6. The reporter assays demonstrated that the regions between − 1219/− 1209 bp and − 1102/− 1092 bp were responsible for STAT3-based miR-506-3p regulation (Fig. 5F and Additional file 1: Figure S3C). Furthermore, in the ChIP assay, we designed two primer set containing two putative STAT3 binding sites to amplify part of the miR-506-3p promoter regions. The results indicated that STAT3 binds to the same site of the promoter of miR-506-3p in both HCT116 (Fig. 5G and H) and HT29 cells (Additional file 1: Figure S3C and S3D). Together, we conclude that the IL6/STAT3 pathway suppresses miR-506-3p transcription in CRC cells after cocultured with TAMs.

**Fig. 6:**
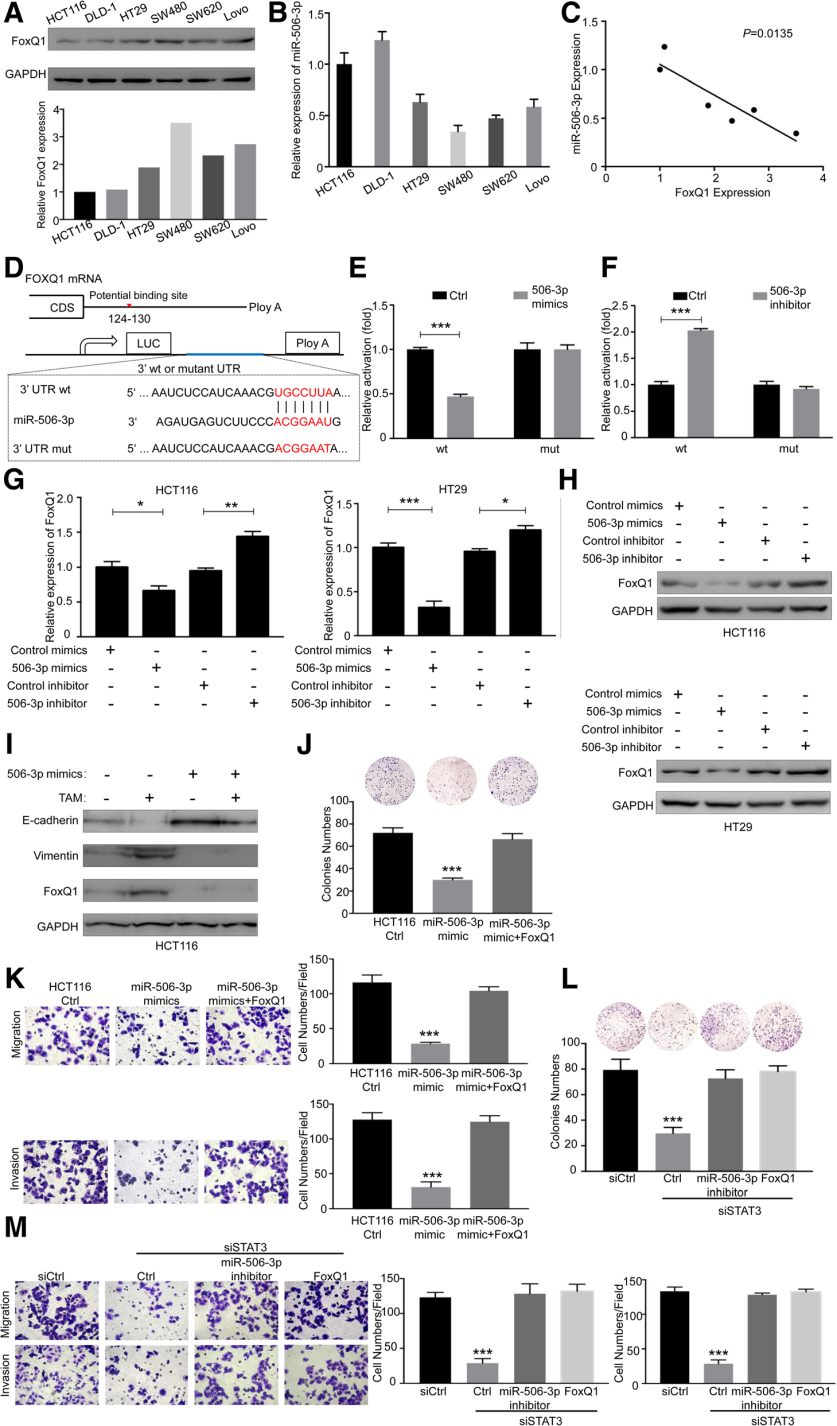
miR-506-3p downregulated FoxQ1 expression by directly binding its 3′UTR, and miR-506-3p/FoxQ1 is essential for STAT3-mediated CRC cell growth, migration, and invasion. (**a**) ﻿Western blot for FoxQ1 in six human CRC cell lines normalized as to expression of GAPDH. (**b**) RT-PCR results of miR-506-3p in indicated CRC cell lines normalized as to expression of U6; Error bars, SD. (**c**) Negative correlation between levels of FoxQ1 and miR-506-3p in CRC cell lines(r = 0.82). (**d**) Schematic representation of the FoxQ1 3′UTR. Mutations were generated at the predicted miR-506-3p–binding sites. (**e**) Luciferase assays demonstrated that expression of FoxQ1 3′UTR (WT or mutant form) by HEK293T cells transfected with miR-506-3p mimics or with control mimics. Error bars, SD. (**f**) Luciferase assays demonstrated that expression of FoxQ1 3′UTR (WT or mutant forms) by HEK293T cells transfected with miR-506-3p inhibitor or with control inhibitor. Error bars, SD. (G) CRC cells (HCT116 and HT29) were transfected with miR-506-3p mimics or inhibitor at a final concentration of 100 and 200 nmol/L, respectively. The levels of FoxQ1 mRNA were analyzed by RT-PCR at 48 h post transfection; Error bars, SEM. (**h**) Levels of FoxQ1 protein were analyzed by Western blot at 72 h post transfection. (**i**) The levels of E-cadherin and Vimentin were analyzed by Western blot at 48 h post miR-506-3p mimics transfection with or without TAMs co-culture. (**j**)﻿ HCT116 cells transfected with miR-506-3p mimics alone or in combination with FoxQ1 and their proliferation were analyzed by colony formation assays. Error bars, SD. (**k**) HCT116 cells transfected with miR-506-3p mimics alone or in combination with FoxQ1 ﻿were subjected to transwell migration and invasion assays. (magnification, × 200). Error bars, SD. (**l**) HCT116 cells transfected with STAT3 siRNA or with control siRNA alone or in combination with miR-506-3p inhibitor or FoxQ1 were subjected to colony formation assays. Error bars, SD. (**m**) HCT116 cells transfected with STAT3 siRNA or with control siRNA alone or in combination with miR-506-3p inhibitor or FoxQ1 were subjected to transwell migration and invasion assays. (magnification, × 200). Error bars, SD. *P < 0.05; **P < 0.01; ***P < 0.001

To confirm whether FoxQ1 was a direct target of miR-506-3p in CRC cells, we measured the levels of miR-506-3p and FoxQ1 in several CRC cell lines by RT-PCR and Western blot (Fig. 6A and B), and found that the endogenous FoxQ1 and miR-506-3p levels were inversely correlated (Fig. 6C). To determine whether miR-506-3p repressed FoxQ1 by targeting the potential binding site, analyses with use of 3′-UTR of luciferase reporter plasmids containing the miR-506-3p target sequences (wt or mutant) on FoxQ1 were performed (Fig. 6D). The overexpression of miR-506-3p suppressed the luciferase activities of the FoxQ1 3′-UTR reporter constructs, whereas the effect was abolished when mutations were introduced into its seed sequences (Fig. 6E). In contrast, inhibition of miR-506-3p increased the luciferase activity in the wt FoxQ1 3′-UTR but not in the mutant form (Fig. 6F). Furthermore, RT-PCR and Western blot revealed that ectopic miR-506-3p expression reduced the mRNA and protein levels of FoxQ1, whereas miR-506-3p knockdown increased FoxQ1 expression (Fig. 6G and H). Collectively, these results indicate that miR-506-3p downregulates FoxQ1 expression by directly binding its 3′UTR.

When HCT116 cells were co-cultured with TAMs, TAMs-induced FoxQ1 expression was largely abrogated by ectopic miR-506-3p expression (Fig. 6I). We have shown that FoxQ1 could induce EMT program above, and the present results suggested that expression of miR-506-3p itself reversed EMT by down-regulation of FoxQ1 (Fig. 6I). Besides the repression of FoxQ1, expression of miR-506-3p itself blocked the effect of TAMs-induced EMT in HCT116 cells (Fig. 6I). Furthermore, we found that the upregulated miR-506-3p expression by its mimics significantly suppressed the proliferative, migration, and invasive capabilities of HCT116 cells, whereas overexpression of FoxQ1 compromised the inhibitory effect mediated by miR-506-3p (Fig. 6J and K).

To evaluate the importance of the STAT3-miR-506-3p-FoxQ1 pathway in CRC progression, we first evaluated the consequences of targeting of STAT3 in cell growth. As anticipated, cell growth of HCT116 cells was significantly decreased with STAT3 knockdown. When miR-506-3p inhibitor or ectopic expression of FoxQ1 was transfected into the STAT3-inhibited HCT116 cells, the proliferative potential of these cells was restored (Fig. 6L). Next, we examined the role of STAT3-miR-506-3p-FoxQ1 pathway in cell migration and invasion, and found that migration and invasion of HCT116 cells were notably blocked after STAT3 knockdown (Fig. 6M). However, miR-506-3p inhibitor or ectopic expression of FoxQ1, respectively, rescued these inhibition effects on migration and invasion (Fig. 6M). Collectively, the above results suggest that the miR-506-3p-FoxQ1 axis is critical for STAT3-induced CRC cell growth, migration, and invasion.

### TAMs enhanced CRC tumorigenesis in vivo

**Fig. 7:**
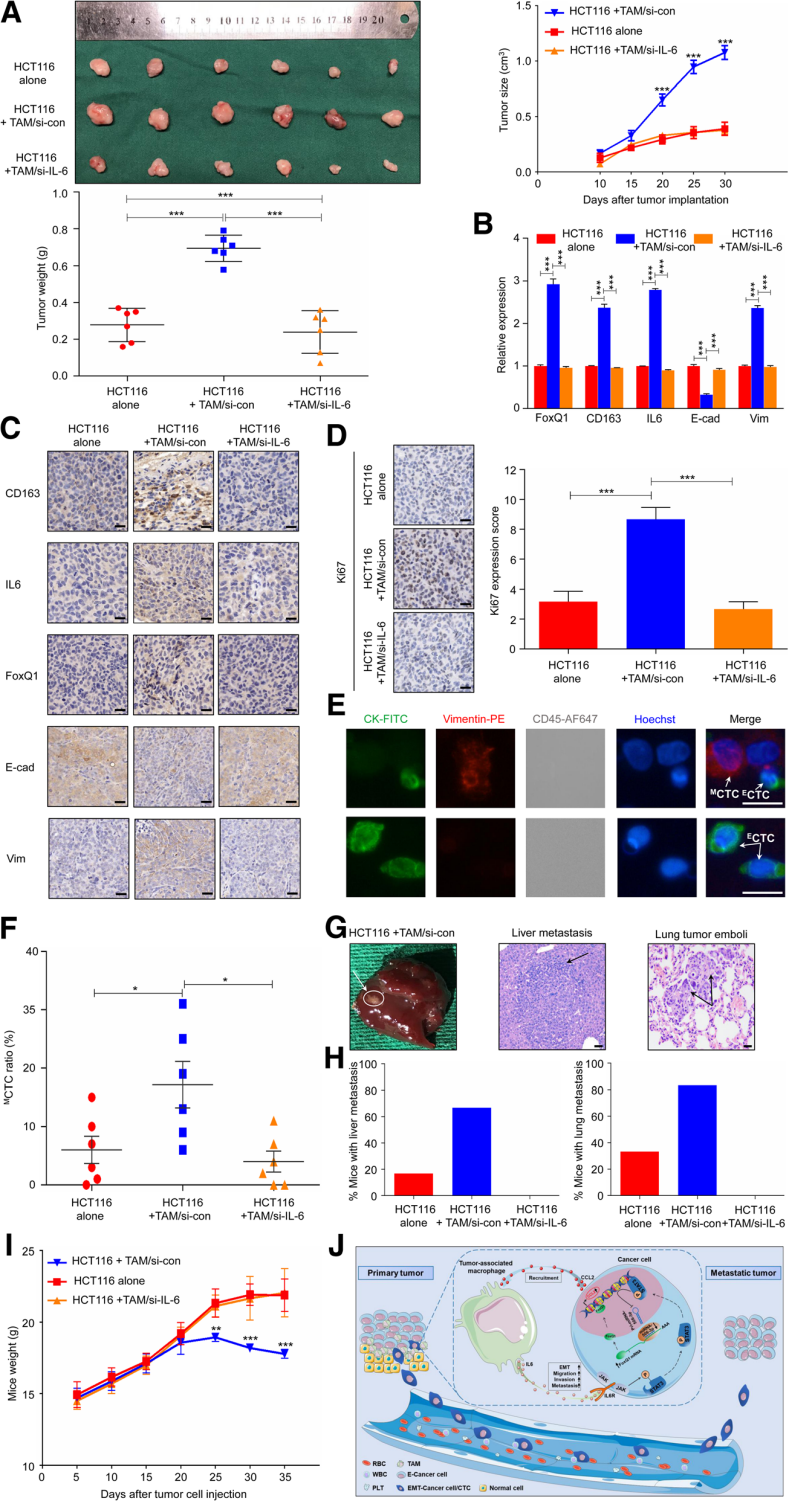
TAMs enhanced CRC tumorigenesis and metastasis in vivo. (**a**) The morphological characteristics of tumor xenograft, tumor size and tumor weight in the HCT116 alone, HCT116 + TAM/si-con and HCT116 + TAM/si-IL6 groups. Error bars, SD. (**b**) The relative expression of FoxQ1, CD163, IL6, E-cadherin and Vimentin mRNA of tumors from the HCT116 alone, HCT116 + TAM/si-con and HCT116 + TAM/si-IL6 groups; Error bars, SD. (**c**) IHC analyzed the expression of FoxQ1, CD163, IL6, E-cadherin and Vimentin protein of tumors from the HCT116 alone, HCT116 + TAM/si-con and HCT116 + TAM/si-IL6 groups; Scale bar, 200×. (**d**) IHC analyzed Ki67 expression in tumor from the HCT116 alone, HCT116 + TAM/si-con and HCT116 + TAM/si-IL6 groups; Scale bar, 200×. (**e**) Representative CTC images from two mice, respectively. Scale bar, 20 μm. (**f**) The ^M^CTC ratio of mice blood from the HCT116 alone, HCT116 + TAM/si-con and HCT116 + TAM/si-IL6 groups. (**g**) Representative images of metastatic lesions in the liver from mice in the HCT116 + TAM/si-con groups at 30 days after implantation, and representative hematoxylin and eosin–stained sections of metastatic nodules in liver and lung are shown. Scale bar, 200×. (**h**) Percentage of mice with metastasis is indicated from HCT116 alone, HCT116 + TAM/si-con and HCT116 + TAM/si-IL6 groups (*n* = 6 per group). (**i**) Weight of nude mice was monitored every 5 days after being injected with HCT116 alone, HCT116 + TAM/si-con and HCT116 + TAM/si-IL6 via the tail veins; (**j**) Schematic illustration of the crosstalk between TAMs and cancer cells in the tumor microenvironment. Our study illustrated a crosstalk between TAMs and cancer cells in the CRC microenvironment. IL6, secreted by TAMs, binds to the IL6 receptor (IL6R) on the cancer cell surface to phosphorylate STAT3. pSTAT3 is translocated to the nucleus to regulate expression of a number of miRNAs, including miR-506-3p, which facilitates the EMT program to mediate ^M^CTC generation, thereby enhancing the migration, invasion, and metastatic potential of CRC cells through miR-506-3p/FoxQ1 axis. In a positive feedback loop, FoxQ1 promoted the secretion of CCL2 from TAMs-educated CRC cells, which increase the recruitment of macrophages. Error bars, SD. *P < 0.05; **P < 0.01; ***P < 0.001

To demonstrate the above in vitro results, an in vivo xenograft model was used. HCT116 cells alone, TAMs alone, HCT116 + TAMs/si-control and HCT116 + TAMs/si-IL6 were injected into the flanks of female nude mice. The tumors produced by co-injection of HCT116 + TAMs/si-control were significantly larger and heavier than those produced by HCT116 alone or HCT116 + TAMs/si-IL6 (Fig. 7A). No tumors were formed following the injection of TAMs alone (data not shown). Moreover, the IHC staining (Fig. 7B) and quantitative data (Fig. 7C) also confirmed the in vitro results that FoxQ1 and STAT3 were significantly elevated in the injection of HCT116 + TAMs/si-control group, accompanied by more CD163^+^ TAMs infiltration and IL6 secretion. Ki-67 staining was also increased in tumors derived from implantation of HCT116 + TAMs/si-control compared with control (Fig. 7D), indicating enhanced proliferation of tumor cells. For malignant metastasis to occur, tumor cells should traverse through the basement membrane and disseminate into the bloodstream. We next examined the presence and ratio of ^M^CTC in three groups. The representative CTC images from two mice were presented in Fig. 7E, and further analysis found the ratio of ^M^CTC was significantly increased in the HCT116 + TAMs/si-control group compared with another two groups (Fig. 7F).

To confirm the effect of TAMs on tumor metastasis in vivo, we injected HCT116 alone, HCT116 + TAMs/si-control, or HCT116 + TAMs/si-IL6 into nude mice via the tail vein. Quantitation of the extent of metastasis by serial sectioning of the livers and lungs of mice in each group revealed livers metastatic lesions in 4 of 6 mice and lungs metastatic lesions in 5 of 6 mice in the HCT116 + TAMs/si-control group (Fig. 7G and H). Moreover, the weight of mice in the HCT116 + TAMs/si-control group was significantly lower than that in the HCT116 cells alone or HCT116 + TAMs/si-IL6 group (Fig. 7I). These results demonstrate that TAMs enhance the growth and metastasis of CRC and IL6 knockdown impaired TAMs-induced CRC tumorigenesis in vivo.

We summarized our findings in a schematic (Fig. 7J). Our study illustrated a crosstalk between TAMs and cancer cells in the CRC microenvironment. IL6, secreted by TAMs, binds to the IL6 receptor (IL6R) on the cancer cell surface to phosphorylate STAT3 (pSTAT3). pSTAT3 is translocated to the nucleus to regulate expression of a number of miRNAs, including miR-506-3p, which facilitates the EMT program to mediate ^M^CTC generation, thereby enhancing the migration, invasion, and metastatic potential of CRC cells through miR-506-3p/FoxQ1 axis. In a positive feedback loop, FoxQ1 promoted the secretion of CCL2 from TAMs-educated CRC cells, which increase the recruitment of macrophages.

## Discussion

In this study, we found that increased CD163^+^ TAMs infiltration in the tumor invasive front was significantly associated with EMT, ^M^CTC ratio and dismal prognosis in CRC. Further studies confirmed that TAMs-derived IL6 induced EMT to enhance migration and invasion of CRC cells by regulating the STAT3/miR-506-3p/FoxQ1 pathway, and elevated CCL2 expression in TAMs-educated CRC cells significantly promoted the recruitment of macrophages in a feedback way.

Clinically, elevated level of CD163^+^ TAMs localized at the invasive front was correlated with EMT phenotype, ^M^CTC ratio, and poor prognosis, indicating their potential roles in facilitating CRC dissemination and invasion. Recently, growing clinical evidences have suggested TAMs and EMT are related. Our results were in accordance with previous study, which comprehensively demonstrated that CCL18^+^ TAMs infiltration in the tumor invasive front might establish an aggressive TME and could regulate breast cancer cells an EMT shift to increase metastatic ability [33]. Lai et al. demonstrated that CD68^+^ TAMs could both decrease Snail expression and inhibit tumor buds which negatively related with EMT phenotype in CRC [34]. Different markers were used to identify TAMs in CRC, and CD68 had been widely recommended as a pan-macrophage marker, making this protein unspecific to the TAMs correlated with tumor growth, which might explain these discrepant effects of TAM subtypes on the EMT regulation of CRC. Furthermore, CTC disseminate from primary tumor by undergoing EMT that allow them to penetrate blood vessels [35], and ^M^CTC was thought to have stronger invasive and metastatic ability [36]. Qi et al. showed high ratio of ^M^CTC prior to resection was significantly associated with early recurrence, multi-intrahepatic recurrence, and lung metastasis in HCC [11]. Our previous study also demonstrated ^M^CTC count in baseline level was significantly correlated with patients’ prognosis in CRC (unpublished data). Currently, our results further found higher ratio of ^M^CTC was detected in peripheral blood of patients with CD163^+^ TAMs infiltrated in invasive front. Tumor invasive front is the most important area for the infiltration of cancer tissues and the immune response of cancerous hosts to cancer. The biological behavior of cancer cells in this location could best reflect the invasive ability of cancer tissues. At present, the clinical associations of high CD163^+^ TAMs infiltration with poor clinical outcomes had been widely shown in numerous human cancers [37, 38], however, whether CD163^+^ TAMs, especially infiltrated in invasive front, contribute to better or poorer prognosis still remains contradictory in terms of CRC [39, 40]. ﻿Herrera, et al. reported that infiltration of CD163^+^ macrophages in CRC tissues was related to the shorter survival time [41]. In contrast, Algars et al. showed that stromal infiltration of CD163^+^ macrophages in CRC was correlated to a significantly improved survival [40]. TAMs are distributed in the different microanatomical locations of CRC tissues, such as tumor center and invasive front, and TAMs in different locations could involve variations with different biological and prognostic properties. Combined the previous and our present results, we therefore supposed that this discrepancy could be the result of macrophages heterogeneity in distinct microanatomical locations, which allowed them to exert antagonistic functions-protumor or antitumor. Above results indicated CD163^+^ TAMs infiltrated in invasive front may promote the production of ^M^CTC by regulating the EMT process of primary tumor cells, thereby affecting tumor progression and prognosis. This was the first study, to our knowledge, where assessment of different TAMs was used in purpose to explore the associations of their sub-localization with EMT phenotype and ratio of ^M^CTC in CRC.

In our study, characterization of in vitro-generated macrophages revealed that HCT116 or HT29-conditioned macrophages exhibited a mixed M1/M2 phenotype, with increased expression of the M2 markers CD163, as well as increased expression of the inflammatory cytokines, IL-1β, IFN-γ, and TNF-α. At present, regarding the roles of TAMs in EMT and tumor metastasis, most studies mainly focused on M2-polarized phenotype [39, 42]. Nevertheless, because polarization of monocytes/macrophages was driven by environmental factors, it was likely that TAMs were not purely polarized M1- or M2- macrophages when facing the plethora of CRC released mediators, but rather exhibited both pro- and anti-inflammatory properties. Indeed, Penny and colleagues also reported that pancreatic ductal adenocarcinoma-generated TAMs expressed both M1 (IL-1β, IL6, and TNF-α) and M2 (CD163, CD206 and Arg1) markers [43]. Moreover, as illustrated by our in vitro co-culture experiments and in vivo animal model, in the presence of mixed phenotype-TAMs, the growth, migration, invasion and metastasis of CRC cells were increased accompanied by EMT phenotype. These results firmly established that mixed phenotype-TAMs, especially with CD163 high expression, was a functional mediator of CRC tumorigenesis in vitro.

Given the key role of cytokines in cell-cell interactions, we screened the changes of a panel of inflammatory cytokines in the TAMs co-cultured with CRC cells, and IL6 was identified as the most significantly upregulated cytokine. Subsequent functional assays confirmed that IL6 was accountable for the TAMs-induced EMT, invasion, and metastasis in CRC. As a key cytokine linked to inflammation-associated cancer, IL6 is implicated in the facilitation of angiogenesis, tumorigenesis and progression by complicated mechanisms, such as increasing expression of invasion-related genes (Twist and MMP-1) and anti-apoptotic factors (Bcl-2 and Bcl-xL), and activation of PI3K, ERK, and STAT3 signaling pathway [44, 45]. Herein, we revealed that IL6 phosphorylated STAT3, which led to EMT of CRC cells. EMT is orchestrated by several transcription factors. Among them, FoxQ1 plays an important role in the invasion and metastasis of many cancers [46, 47]. Our study demonstrated FoxQ1 was the most upregulated transcription factors in CRC cells cocultured with TAMs, and silencing FoxQ1 abrogated TAMs-mediated EMT change and invasion/metastasis, indicating a driving role of FoxQ1 in the TAMs-induced EMT and aggressiveness of CRC. Consistently, Guo et al. reported that FoxQ1 was essential for TAMs-induced EMT and metastasis in gastric cancer cells [48]. Notably, our study also found a novel reciprocal activation between cancer cells and TAMs that FoxQ1 expression in CRC cells co-cultured with TAMs promoted macrophage attraction in a CCL2-dependent manner. Previously, a study also reported FoxQ1 expression could promote macrophage infiltration through the VersicanV1/CCL2 axis in HCC [31]. Additionally, Wolf et al. showed that CCL2 produced by CRC cells could also foster vascularization and intravasation [49]. In breast cancer, inflammatory monocytes could be continually recruit by CCL2 produced by cancer cells and differentiate into TAMs that facilitate the subsequent growth of metastatic cells [50]. At present, our group is conducting an in-depth study to explore the specific role of recruited macrophages in the CRC microenvironment. On the basis of our data and previous studies, we proposed that FoxQ1 expressed in cancer cells was closely involved in development of the TME by inducing invasion, metastasis, and chemotactic activity.

MiRNAs are small non-coding RNAs that affect tumor progression, achieved by binding to the target gene. Here, we found an inverse correlation between miR-506-3p and FoxQ1 expression in CRC cell lines, and further proved miR-506-3p targets FoxQ1 mRNA by binding to the FoxQ1 3’UTR to inhibit TAMs-induced EMT of CRC cells. The results of our study were consistent with the finding in previous reporters that miR-506-3p could significantly inhibit cell growth, invasion and enhance the chemotherapeutic response in CRC [51, 52]. In the present study, we also found that FoxQ1 expression was regulated by STAT3, but this regulation was indirect and involved miR-506-3p as an intermediary, which indicated a potential link between FoxQ1 and IL6/STAT3 signaling. STAT3 directly bound two sites in the miR-506-3p promoter regions and was required for transcriptional regulation of this miRNA. STAT3-induced transcription of protein-coding genes has been widely reported in various types of cancers; however, the role of STAT3 in the transcription of non-coding genes, such as miRNAs, is relatively less studied. Recent results found that STAT3 played an important role in miRNA regulation. For example, STAT3 activated by IL6 directly upregulated the miR-21 and miR-181b-1, which was shown to be necessary for the epigenetic switch linking inflammation to cancer [53]. Furthermore, STAT3-mediated activation of miR-182-5p had been shown to upregulate the proliferative and invasive capacities by directly targeting PCDH8 in glioma cells [54]. STAT3-repressed miR-34a were also required for invasion and metastasis of human CRC cells [55]. These data cumulatively demonstrated that STAT3-miRNAs interactions were emerging as key regulators of the malignant phenotype of cancer cells. Moreover, we also provided strong evidence that the activation of FoxQ1 induced by STAT3/miR-506-3p was important for CRC cell growth, migration, and invasion, which indicated that STAT3-miR-506-3p-FoxQ1 signal axis played an imperative role in the TAMs-medicated CRC progression.

## Conclusion

In summary, our current findings disclose an important mechanism which involves a positive feedback loop between TAMs and cancer cells, and this mechanism is essential to the initiation, progression and metastasis of CRC. Our study highlights that IL6/STAT3/miR-506-3p/FoxQ1 signal cascade might be a potential therapeutic target for combating CRC.

## Additional file

Additional file 1: **Table S1.** Correlation between the density of macrophages at non-invasive front and clinicopathologic parameters (*n* = 81). **Table S2.** Potential STAT3 binding site on miR-506-3p promoter. **Table S3.** The sequences of the primers for quantitative RT-PCR. **Figure S1.** CD68^+^ and CD163^+^ TAMs at non-invasive front are not associated with EMT, ^M^CTC ratio, and poor prognosis in CRC patients. (A-B) Expression of E-cadherin and Vimentin in human CRC samples with low or high CD68 and CD163 expression at non-invasive front, respectively. (C-D) Correlation analysis between CD68 expression and CD163 at non-invasive front and ^M^CTC ratio, respectively. (E-F) CD68 expression at non-invasive front and the patients’ recurrence-free survival and overall survival in CRC, respectively. (G-H) CD163 expression at non-invasive front and the patients’ recurrence-free survival and overall survival in CRC, respectively. Error bars, SEM. ns, not significant. **Figure S2.** Validation of in-vitro-generated TAMs. (A) Flow cytometry for analyzing the expression of HLA-DR, CD80, CD206, and CD163 in PMA-treated THP-1 macrophages incubated with conditioned media (CM) from CRC cell lines (HT-29 or HCT116) or normal cell line (NCM460) for 48 h. (B) ELISA for analyzing the secretion of IL-10 and IL-12 in PMA-treated THP-1 macrophages incubated with conditioned media (CM) from CRC cell lines (HT-29 or HCT116) or normal cell line (NCM460) for 48 h; Error bars, SD. (C) ELISA for analyzing the secretion of IL-1β, IFN-γ, and TNF-α in PMA-treated THP-1 macrophages incubated with conditioned media (CM) from CRC cell lines (HT-29 or HCT116) or normal cell line (NCM460) for 48 h; Error bars, SD. (D) The morphology of colorectal cancer cells (HCT116 and HT29) with or without TAMs-coculture. ns, not significant; *P < 0.05; **P < 0.01; ***P < 0.001. **Figure S3.** STAT3 directly suppressed miR-506-3p expression in CRC cells. (A) A graphical illustration of five potential STAT3 transcriptional factor binding sites in the miR-506-3p promoter region. (B) Serially truncated and mutated miR-506-3p promoter constructs were cloned to pGL3-Basic luciferase reporters and transfected into HT29 cells. The relative luciferase activities were determined after IL-6 (50 ng/mL) treatment for 1 h; Error bars, SD. (C) Selective mutation analyses identified STAT3-responsive regions in the miR-506-3p promoter in HT29 cells; Error bars, SD. (D) ChIP assay demonstrated the direct binding of STAT3 to the miR-506-3p promoter, including nonspecific control (N.C), CHIP1, and CHIP2 in HT29 cells. Input, 5% of total lysate. (E) RT-PCR of the ChIP products confirmed the direct binding capacity of STAT3 to the miR-506-3p promoter in HT29 cells. Input, 5% of total lysate; Error bars, SD. ns, not significant; *P < 0.05; **P < 0.01; ***P < 0.001 (DOCX 2752 kb)

## Abbreviations

3’UTR: 3′ untranslated region
CCL2: (C-C motif) ligand 2
CRC: colorectal cancer
CTC: circulating tumor cell
^E^CTC: epithelial circulating tumor cell
EMT: epithelial-mesenchymal transition
FoxQ1: forkhead box Q1
IHC: immunohistochemistry
IL6: interleukin-6
IRS: immunoreactive score
^M^CTC: mesenchymal circulating tumor cell
miR: microRNA
PP: percentage of positive cells
SI: staining intensity
STAT3: signal transducer and activator of transcription 3
TAMs: tumor-associated macrophages
TME: tumor microenvironment
WBC: white blood cell
