# Supplementary material for: Crosstalk between cancer cells and tumor associated macrophages is required for mesenchymal circulating tumor cell-mediated colorectal cancer metastasis
Source: Mol Cancer. 2019 Mar 30;18:64. doi: 10.1186/s12943-019-0976-4 (PMC6441214; doi:10.1186/s12943-019-0976-4)
Supplement: Supplementary file 1 — Table S1. Correlation between the density of macrophages at non-invasive front and clinicopathologic parameters (n = 81). Table S2. Potential STAT3 binding site on miR-506-3p promoter. Table S3. The sequences of the primers for quantitative RT-PCR. Figure S1. CD68+ and CD163+ TAMs at non-invasive front are not associated with EMT, MCTC ratio, and poor prognosis in CRC patients. (A-B) Expression of E-cadherin and Vimentin in human CRC samples with low or high CD68 and CD163 expression at non-invasive front, respectively. (C-D) Correlation analysis between CD68 expression and CD163 at non-invasive front and MCTC ratio, respectively. (E-F) CD68 expression at non-invasive front and the patients’ recurrence-free survival and overall survival in CRC, respectively. (G-H) CD163 expression at non-invasive front and the patients’ recurrence-free survival and overall survival in CRC, respectively. Error bars, SEM. ns, not significant. Figure S2. Validation of in-vitro-generated TAMs. (A) Flow cytometry for analyzing the expression of HLA-DR, CD80, CD206, and CD163 in PMA-treated THP-1 macrophages incubated with conditioned media (CM) from CRC cell lines (HT-29 or HCT116) or normal cell line (NCM460) for 48 h. (B) ELISA for analyzing the secretion of IL-10 and IL-12 in PMA-treated THP-1 macrophages incubated with conditioned media (CM) from CRC cell lines (HT-29 or HCT116) or normal cell line (NCM460) for 48 h; Error bars, SD. (C) ELISA for analyzing the secretion of IL-1β, IFN-γ, and TNF-α in PMA-treated THP-1 macrophages incubated with conditioned media (CM) from CRC cell lines (HT-29 or HCT116) or normal cell line (NCM460) for 48 h; Error bars, SD. (D) The morphology of colorectal cancer cells (HCT116 and HT29) with or without TAMs-coculture. ns, not significant; *P < 0.05; **P < 0.01; ***P < 0.001. Figure S3. STAT3 directly suppressed miR-506-3p expression in CRC cells. (A) A graphical illustration of five potential STAT3 transcriptional factor binding sites in the miR-506- [file 12943_2019_976_MOESM1_ESM.docx]

**Crosstalk between cancer cells and tumor associated macrophages is required for mesenchymal circulating tumor cell-mediated colorectal cancer metastasis**

Chen Wei^1,2,3†^, Chaogang Yang^1,2,3†^, Shuyi Wang^1,2,3†^, Dongdong Shi^1,2,3^, Chunxiao Zhang^1,2,3^, Xiaobin Lin^1,2,3^, Qing Liu^1,2,3^, Rongzhang Dou^1,2,3^, Bin Xiong^1,2,3*^

^1^Department of Gastrointestinal Surgery & Department of Gastric and Colorectal Surgical Oncology, Zhongnan Hospital of Wuhan University, ^2^Hubei Key Laboratory of Tumor Biological Behaviors, ^3^Hubei Cancer Clinical Study Center, Wuhan 430071, People’s Republic of China

^†^These authors contributed equally to this work.

^*^Correspondence: [binxiong1961@whu.edu.cn](mailto:binxiong1961@whu.edu.cn)

Email addresses for all authors: Chen Wei (weic@whu.edu.cn)

Chaogang Yang ([ycg@whu.edu.cn](mailto:ycg@whu.edu.cn))

Shuyi Wang (shuyiwang@whu.edu.cn)

Dongdong Shi (dondongpds@163.com)

Chunxiao Zhang (zhang_chunxiao@163.com)

Xiaobin Lin (dondongpds@163.com)

Qing Liu (liuqing900108@163.com)

Rongzhang Dou (dourongzhang@126.com)

Bin Xiong ([binxiong1961@whu.edu.cn](mailto:binxiong1961@whu.edu.cn))

| **Table S1 Correlation between the density of macrophages at non-invasive front and clinicopathologic parameters (n = 81)** | | | | | | | | | | | | | | |
| --- | --- | --- | --- | --- | --- | --- | --- | --- | --- | --- | --- | --- | --- | --- |
| Parameters | | n (%) | | CD68 expression | | | | |  | | CD163 expression | | |  |
|  |  |  |  | Low | | High | *P* | |  | | Low | High | *P* |  |
| Gender | |  | |  | |  |  | |  | |  |  |  |  |
| Male | | 48 (59.3) | | 22 | | 26 | 0.441 | |  | | 22 | 18 | 0.315 |  |
| Female | | 33 (40.7) | | 18 | | 15 |  | |  | | 18 | 23 |  |  |
| Age, years | |  | |  | |  |  | |  | |  |  |  |  |
| ＜60 | | 39 (48.1) | | 20 | | 19 | 0.742 | |  | | 21 | 18 | 0.508 |  |
| ≥60 | | 42 (51.9) | | 20 | | 22 |  | |  | | 19 | 23 |  |  |
| Tumor site | |  | |  | |  |  | |  | |  |  |  |  |
| Colon | | 42(51.9) | | 17 | | 25 | 0.096 | |  | | 24 | 19 | 0.218 |  |
| Rectal | | 39 (48.1) | | 23 | | 16 |  | |  | | 16 | 22 |  |  |
| Tumor size, cm | |  | |  | |  |  | |  | |  |  |  |  |
| ＜5 | | 54 (66.7) | | 24 | | 30 | 0.209 | |  | | 30 | 24 | 0.116 |  |
| ≥5 | | 27 (33.3) | | 16 | | 11 |  | |  | | 10 | 17 |  |  |
| Tumor rade | |  | |  | |  |  | |  | |  |  |  |  |
| Poor | | 32 (39.5) | | 18 | | 14 | 0.318 | |  | | 11 | 21 | **0.029** |  |
| Moderate/Well | | 49(60.5) | | 22 | | 27 |  | |  | | 29 | 20 |  |  |
| LVI | |  | |  | |  |  | |  | |  |  |  |  |
| Absence | | 45(55.6) | | 24 | | 21 | 0.427 | |  | | 16 | 23 | 0.147 |  |
| Presence | | 36(44.4) | | 16 | | 20 |  | |  | | 24 | 18 |  |  |
| PNI | |  | |  | |  |  | |  | |  |  |  |  |
| Absence | | 43(53.1) | | 23 | | 20 | 0.432 | |  | | 23 | 20 | 0.432 |  |
| Presence | | 38(46.9) | | 17 | | 21 |  | |  | | 17 | 21 |  |  |
| TI | |  | |  | |  |  | |  | |  |  |  |  |
| T1-2 | | 12(14.8) | | 7 | | 5 | 0.502 | |  | | 8 | 6 | 0.523 |  |
| T3-4 | | 69(85.2) | | 33 | | 36 |  | |  | | 32 | 35 |  |  |
| LNM | |  | |  | |  |  | |  | |  |  |  |  |
| N0-1 | | 53(65.4) | | 23 | | 31 | 0.084 | |  | | 30 | 23 | 0.074 |  |
| N2-3 | | 28(34.6) | | 17 | | 10 |  | |  | | 10 | 18 |  |  |
| TNM stage^a^ | |  | |  | |  |  | |  | |  |  |  |  |
| I/II | | 42(51.9) | | 18 | | 23 | 0.318 | |  | | 24 | 18 | 0.147 |  |
| III | | 39(48.1) | | 22 | | 18 |  | |  | | 16 | 23 |  |  |
| CA19-9, U/mL | |  | |  | |  |  | |  | |  |  |  |  |
| ＜37 | | 57(70.4) | | 32 | | 25 | 0.061 | |  | | 27 | 30 | 0.576 |  |
| ≥37 | | 24(29.6) | | 8 | | 16 |  | |  | | 13 | 11 |  |  |
| CEA, ng/ml | |  | |  | |  |  | |  | |  |  |  |  |
| ＜5 | | 55(67.9) | | 29 | | 26 | 0.381 | |  | | 28 | 27 | 0.689 |  |
| ≥5 | | 26(32.1) | | 11 | | 15 |  | |  | | 12 | 14 |  |  |
| Overall | | 81(100.0) | | 40 | | 41 |  | |  | | 40 | 41 |  |  |
| Notes: ^a^The 8th edition of the AJCC Cancer Staging Manual. Abbreviations: LVI, lymphovascular invasion; PNI, perineural invasion; TI, tumor invasion; LNM, lymph node metastasis; TNM, tumor-node-metastasis; CA19-9, carbohydrate antigen 19-9; CEA, carcinoembryonic antigen; CD68, cluster of differentiation 68; CD163, cluster of differentiation 163. | | | | | | | | | | | | | |  |
| **Table S2** Potential STAT3 binding site on miR-506-3p promoter | | | | | | | | | | | | | | |
| **Score** | **Relative score** | | **Start** | | **End** | | | **Strand** | | **Predicted site sequence** | | | | |
| 9.358 | 0.910928052555 | | 757 | | 767 | | | 1 | | CTGTTTGGAAT | | | | |
| 8.219 | 0.897130015136 | | 1092 | | 1102 | | | 1 | | GTTCTTAAAAA | | | | |
| 6.905 | 0.881216567093 | | 1322 | | 1332 | | | -1 | | AATCTGGAAAA | | | | |
| 6.492 | 0.876204826771 | | 1860 | | 1870 | | | 1 | | CTACTAGAAAA | | | | |
| 6.475 | 0.876006479044 | | 1209 | | 1219 | | | 1 | | CTCCCTGAAAG | | | | |

| **Table S3** The sequences of the primers for quantitative RT-PCR | | |
| --- | --- | --- |
| Gene | ﻿Primer Sequence (5′ to 3′) | Product size (bp) |
| Arginase 1 | F: TGGACAGACTAGGAATTGGCA  R: CCAGTCCGTCAACATCAAAACT | 102 |
| CD163 | F: TTTGTCAACTTGAGTCCCTTCAC  R: TCCCGCTACACTTGTTTTCAC | 127 |
| CD206 | F: GGGTTGCTATCACTCTCTATGC  R: TTTCTTGTCTGTTGCCGTAGTT | 126 |
| CD68 | F: CTTCTCTCATTCCCCTATGGACA  R: GAAGGACACATTGTACTCCACC | 105 |
| CD86 | F: CTGCTCATCTATACACGGTTACC  R: GGAAACGTCGTACAGTTCTGTG | 133 |
| HLA-DR | F: TCTGGCGGCTTGAAGAATTTG  R: GGTGATCGGAGTATAGTTGGAGC | 125 |
| E-cadherin | F: ATTTTTCCCTCGACACCCGAT  R: TCCCAGGCGTAGACCAAGA | 109 |
| Vimentin | F: AGTCCACTGAGTACCGGAGAC  R: CATTTCACGCATCTGGCGTTC | 98 |
| TNF-α | F: TCTCGAACCCCGAGTGACAA  R: TGAAGAGGACCTGGGAGTAG | 181 |
| EGF | F: TCTGAATGTCCCCTGTCCCACG  R: CTGCGACTCCTCACATCTCTGC | 326 |
| IL-8 | F: AAACCACCGGAAGGAACCAT  R: CCTTCACACAGAGCTGCAGAAA | 101 |
| TGF-β | F: AAGGACCTCGGCTGGAAGTGC  R: CCGGGTTATGCTGGTTGTA | 136 |
| CCL2 | F: AAGATCTCAGTGCAGAGGCTCG  R: CACAGATCTCCTTGGCCACAA | 103 |
| IL-6 | F: AATAACCACCCCTGACCCAAC  R: ACATTTGCCGAAGAGCCCT | 149 |
| IL-1β | F: TGAACTGAAAGCTCTCCACC  R: CTGATGTACCAGTTGGGGAA | 297 |
| CCL18 | F: CTCTGCTGCCTCGTCTATACCT  R: CTTGGTTAGGAGGATGACACCT | 108 |
| HGF | F: GCTATCGGGGTAAAGACCTACA  R: CGTAGCGTACCTCTGGATTGC | 99 |
| STAT3 | F: ACCAGCAGTATAGCCGCTTC  R: GCCACAATCCGGGCAATCT | 124 |
| TWIST1 | F: CACTGAAAGGAAAGGCATCA  R: GGCCAGTTTGATCCCAGTAT | 108 |
| HMGA2 | F: ACCCAGGGGAAGACCCAAA  R: CCTCTTGGCCGTTTTTCTCCA | 93 |
| FoxQ1 | F: CACGCAGCAAGCCATATACG  R: CGTTGAGCGAAAGGTTGTGG | 198 |
| Snail2 | F: CGAACTGGACACACATACAGTG  R: CTGAGGATCTCTGGTTGTGGT | 87 |
| Snail1 | F: TCGGAAGCCTAACTACAGCGA  R: AGATGAGCATTGGCAGCGAG | 140 |
| PRX1 | F: CCCGGATGCTTTTGTTCGAGA  R: CATGTGGCAGAATAAGTAGCCAT | 273 |
| ZEB2 | F: AGGAGCAGGTAATCG  R: TGGGCACTCGTAAGG | 115 |
| AP4 | F: GAGGGCTCTGTAGCCTTGC  R: GAATCCCGCGTTGATGCTCT | 128 |
| ZEB1 | F: CGAGTCAGATGCAGAAAATGAGCAA  R: ACCCAGACTGCGTCACATGTCTT | 371 |
| miR-422a | RT:GTCGTATCCAGTGCGTGTCGTGGAGTCGGCAATTGCACTGGATACGACGCCTTCTG  F: GGGTCAGAAGGCGTCGT | 53 |
| miR-320a | RT:GTCGTATCCAGTGCGTGTCGTGGAGTCGGCAATTGCACTGGATACGACGGAAGAAC  F: GCCTTCTCTTCCCGGTTCT | 63 |
| miR-320b | RT:GTCGTATCCAGTGCGTGTCGTGGAGTCGGCAATTGCACTGGATACGACTTGCCCTC  F: GCTGGGTTGAGAGGGCA | 59 |
| miR-133b | RT:GTCGTATCCAGTGCGTGTCGTGGAGTCGGCAATTGCACTGGATACGACTAGCTGGT  F: GTCCCCTTCAACCAGCTAGT | 59 |
| miR-96-5p | RT:GTCGTATCCAGTGCGTGTCGTGGAGTCGGCAATTGCACTGGATACGACAGCAAAAA  F: GCCGAGGGCACUAGCACAUU | 62 |
| miR-128-3p | RT:GTCGTATCCAGTGCGTGTCGTGGAGTCGGCAATTGCACTGGATACGACAAAGAGAC  F: TCACAGTGAACCGGTCTCT | 62 |
| miR-506-3p | RT:GTCGTATCCAGTGCGTGTCGTGGAGTCGGCAATTGCACTGGATACGACTCTACTCA  F: TCTGAGTAGAGTCGTATCCAGT | 51 |
| miR-320c | RT:GTCGTATCCAGTGCGTGTCGTGGAGTCGGCAATTGCACTGGATACGACACCCTCTC  F: GCTGGGTTGAGAGGGTGT | 57 |
| miR-320d | RT:GTCGTATCCAGTGCGTGTCGTGGAGTCGGCAATTGCACTGGATACGACTCCTCTCA  F: CTGGGTTGAGAGGAGTCGT | 55 |
| miR-1271-5p | RT:GTCGTATCCAGTGCGTGTCGTGGAGTCGGCAATTGCACTGGATACGACTGAGTGCT  F: CCTAGCAAGCACTCAGTCG | 56 |
| Universal downstream | R: CAGTGCGTGTCGTGGAGT |  |
| GAPDH | F: GCACCACCAACTGCTTAGCA  R: GTCTTCTGGGTGGCAGTGATG | 106 |
| U6 | F: CTCGCTTCGGCAGCACA  R: AACGCTTCACGAATTTGCGT | 96 |

**
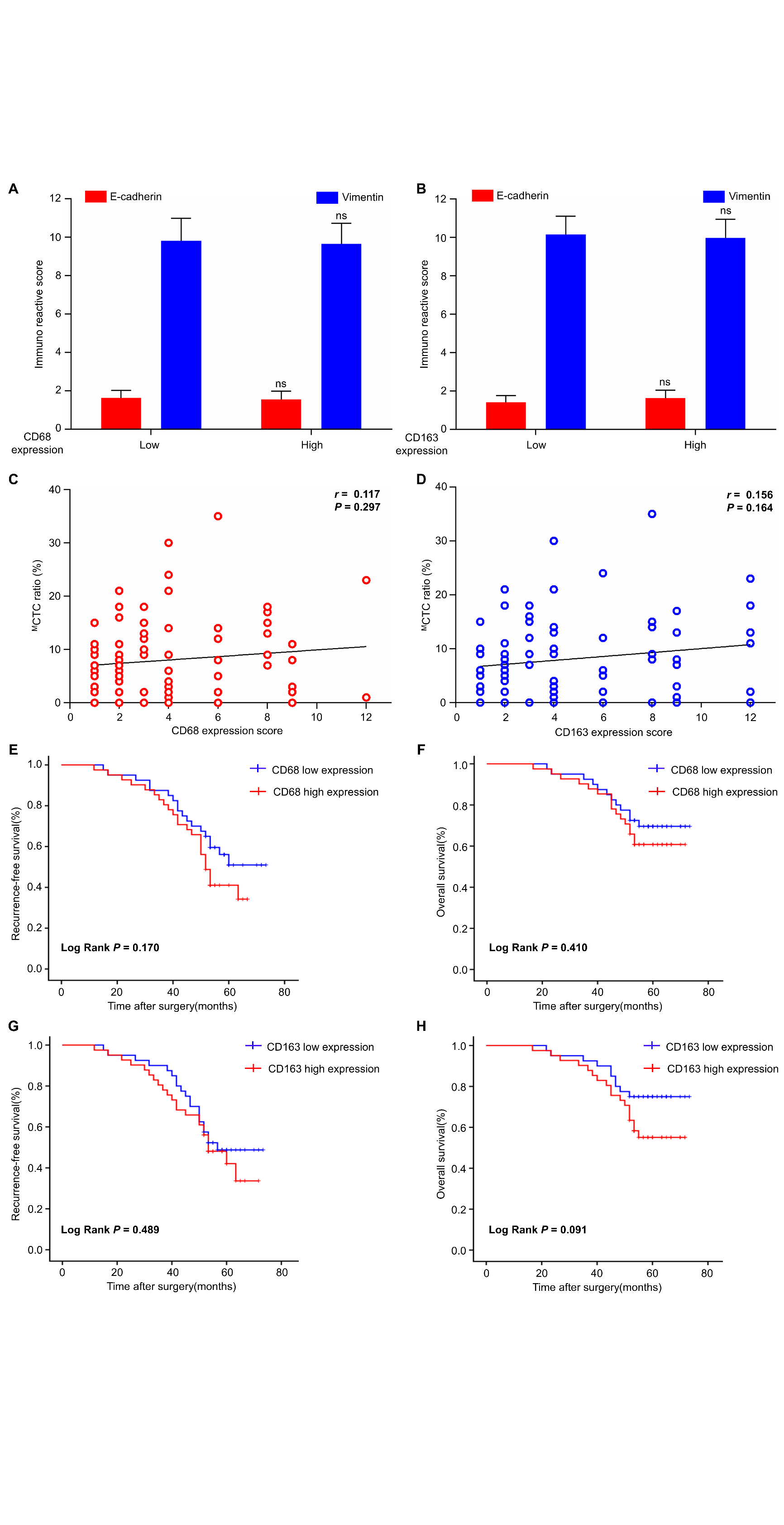
**

**Fig. S1** CD68^+^ and CD163^+^ TAMs at non-invasive front are not associated with EMT, ^M^CTC ratio, and poor prognosis in CRC patients. (A-B) Expression of E-cadherin and Vimentin in human CRC samples with low or high CD68 and CD163 expression at non-invasive front, respectively. (C-D) Correlation analysis between CD68 expression and CD163 at non-invasive front and ^M^CTC ratio, respectively. (E-F) CD68 expression at non-invasive front and the patients’ recurrence-free survival and overall survival in CRC, respectively. (G-H) CD163 expression at non-invasive front and the patients’ recurrence-free survival and overall survival in CRC, respectively. Error bars, SEM. ns, not significant.

**
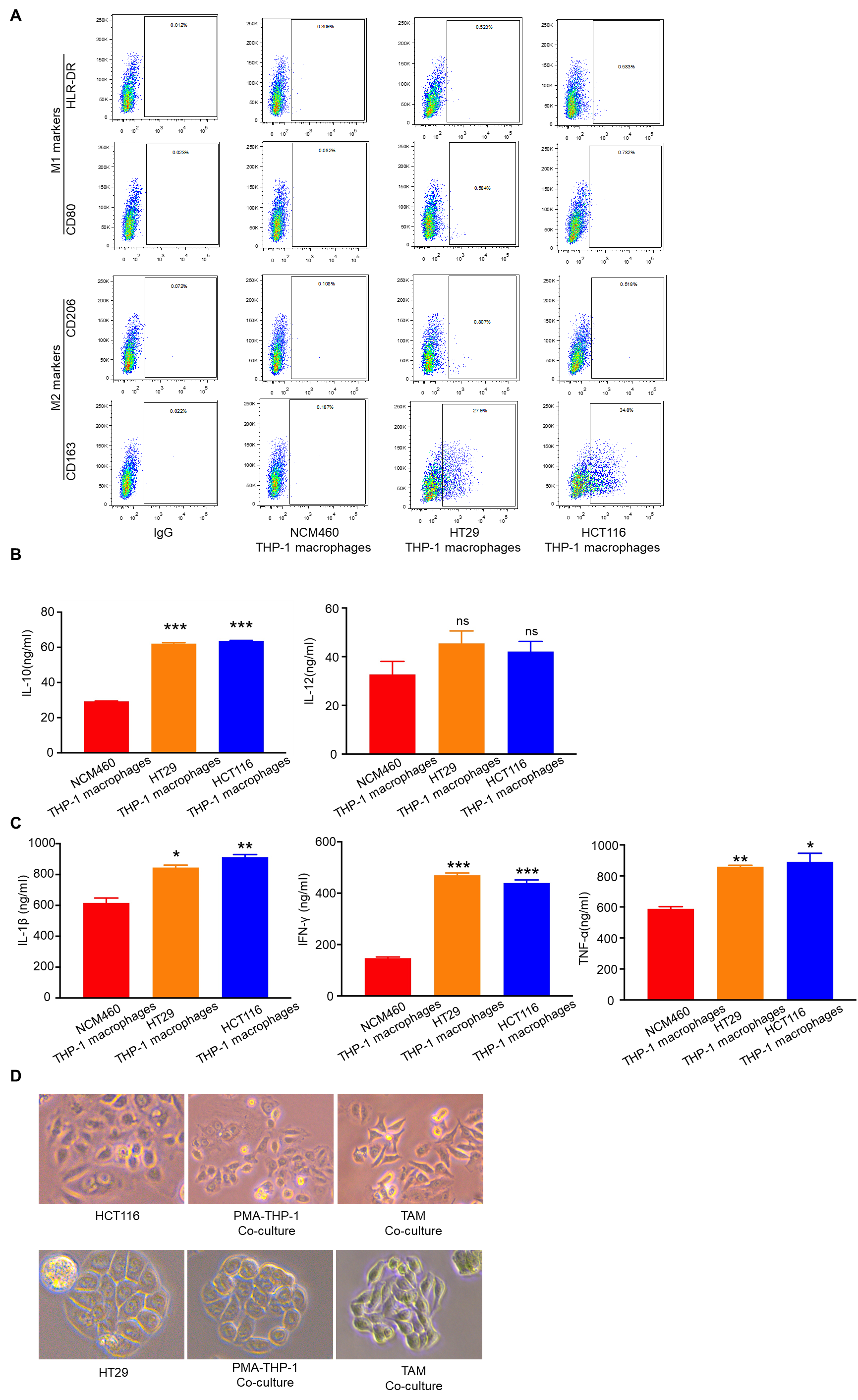
**

**Fig. S2** **﻿** Validation of in-vitro-generated TAMs. (A) Flow cytometry for analyzing the expression of HLA-DR, CD80, CD206, and CD163 in PMA-treated THP-1 macrophages incubated with conditioned media (CM) from CRC cell lines (HT-29 or HCT116) or normal cell line (NCM460) for 48h. (B) ELISA for analyzing the secretion of IL-10 and IL-12 in PMA-treated THP-1 macrophages incubated with conditioned media (CM) from CRC cell lines (HT-29 or HCT116) or normal cell line (NCM460) for 48h; Error bars, SD. (C) ELISA for analyzing the secretion of IL-1β, IFN-γ, and TNF-α in PMA-treated THP-1 macrophages incubated with conditioned media (CM) from CRC cell lines (HT-29 or HCT116) or normal cell line (NCM460) for 48h; Error bars, SD. (D) The morphology of colorectal cancer cells (HCT116 and HT29) with or without TAMs-coculture. ns, not significant; **P*<0.05; ***P*<0.01; ****P*<0.001.


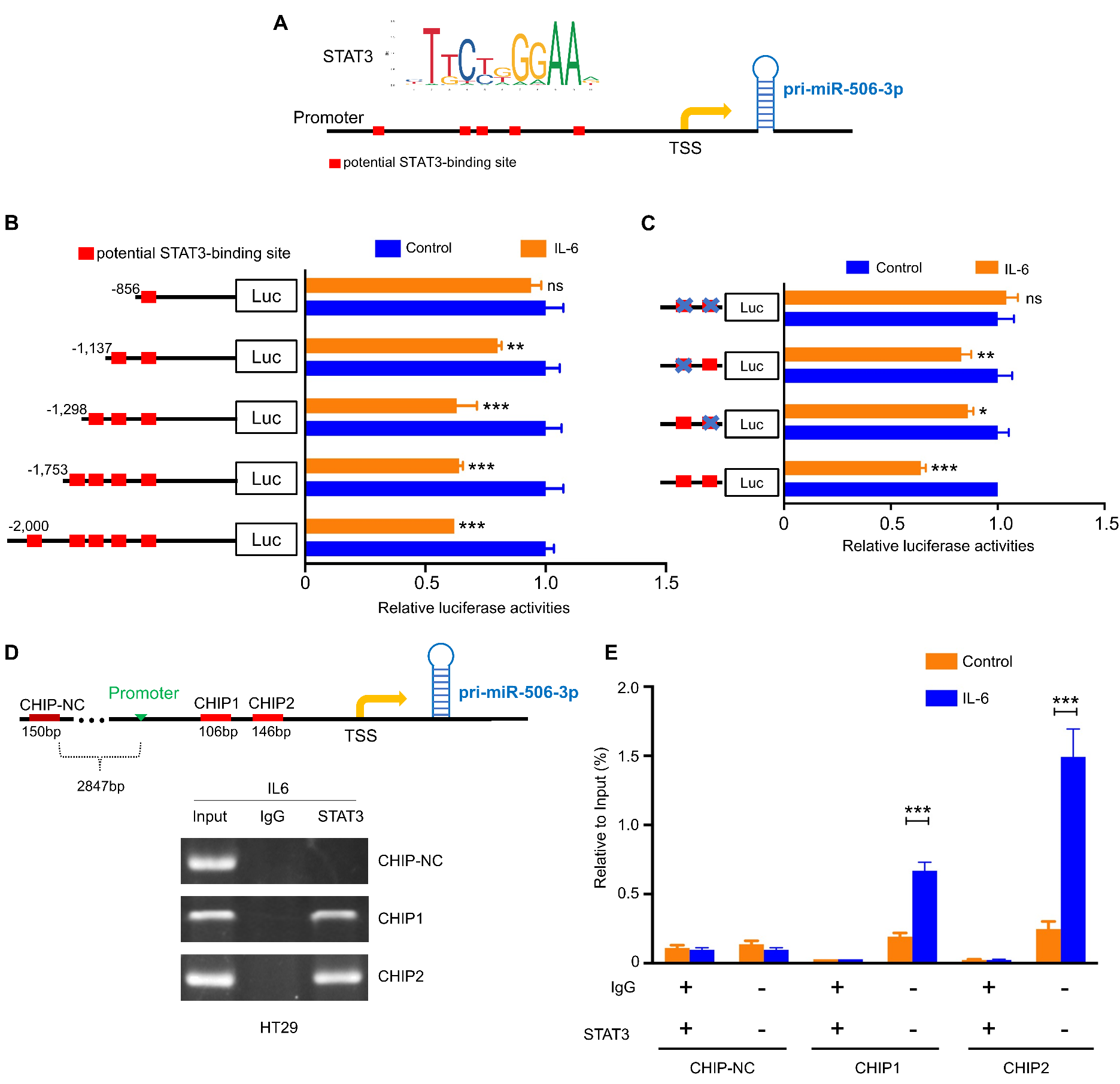


**Fig. S3** STAT3 directly suppressed miR-506-3p expression in CRC cells. (A) A graphical illustration of five potential STAT3 transcriptional factor binding sites in the miR-506-3p promoter region. (B) Serially truncated and mutated miR-506-3p promoter constructs were cloned to pGL3-Basic luciferase reporters and transfected into HT29 cells. The relative luciferase activities were determined after IL-6 (50 ng/mL) treatment for 1h; Error bars, SD. (C) Selective mutation analyses identified STAT3-responsive regions in the miR-506-3p promoter in HT29 cells; Error bars, SD. (D) ChIP assay demonstrated the direct binding of STAT3 to the miR-506-3p promoter, including nonspecific control (N.C), CHIP1, and CHIP2 in HT29 cells. Input, 5% of total lysate. (E) RT-PCR of the ChIP products confirmed the direct binding capacity of STAT3 to the miR-506-3p promoter in HT29 cells. Input, 5% of total lysate; Error bars, SD. ns, not significant; **P*<0.05; ***P*<0.01; ****P*<0.001.
